# Fe-doped chrysotile nanotubes containing siRNAs to silence *SPAG5* to treat bladder cancer

**DOI:** 10.1186/s12951-021-00935-z

**Published:** 2021-06-23

**Authors:** Jianye Liu, Yi Zhang, Hongliang Zeng, Long Wang, Qun Zhang, Pei Wu, Xiaoming Liu, Hongyi Xie, Wei Xiang, Biao Liu, Jiahao Liu, Xuewen Liu, Jianfei Xie, Jin Tang, Zhi Long, Leye He, Mengqing Xiao, Liang Xiang, Ke Cao

**Affiliations:** 1 Department of Urology, The Third Xiangya Hospital of Central South University, Changsha, 410013 China; 2 School of Minerals Processing and Bioengineering, Central South University, Changsha, 410083 China; 3 Research Institute of Chinese Medicine, Hunan Academy of Chinese Medicine, Changsha, 410013 China; 4 Department of Radiotherapy, The First Affiliated Hospital of Sun Yat-Sen University, Guangzhou, 510080 China; 5 Department of Operation Center, The Second Xiangya Hospital of Central South University, Changsha, 410008 China; 6 Department of Digestive, The Third Xiangya Hospital of Central South University, Changsha, 410013 China; 7 Department of Oncology, The Third Xiangya Hospital of Central South University, No.138, Tongzipo Road, Changsha, 410013 Hunan China; 8 Department of Nursing, The Third Xiangya Hospital of Central South University, Changsha, 410013 China

**Keywords:** Fe-doped chrysotile nanotubes, Gene therapy, Targeted delivery, SiRNA-SPAG5, Bladder cancer

## Abstract

**Background:**

For certain human cancers, sperm associated antigen 5 (SPAG5) exerts important functions for their development and progression. However, whether RNA interference (RNAi) targeting *SPAG5* has antitumor effects has not been determined clinically.

**Results:**

The results indicated that Fe-doped chrysotile nanotubes (FeSiNTs) with a relatively uniform outer diameter (15–25 nm) and inner diameter (7–8 nm), and a length of several hundred nanometers, which delivered an siRNA against the *SPAG5* oncogene (siSPAG5) efficiently. The nanomaterials were designed to prolong the half-life of siSPAG5 in blood, increase tumor cell-specific uptake, and maximize the efficiency of *SPAG5* silencing. In vitro, FeSiNTs carrying siSPAG5 inhibited the growth, migration, and invasion of bladder cancer cells. In vivo, the FeSiNTs inhibited growth and metastasis in three models of bladder tumors (a tail vein injection lung metastatic model, an in-situ bladder cancer model, and a subcutaneous model) with no obvious toxicities. Mechanistically, we showed that FeSiNTs/siSPAG5 repressed PI3K/AKT/mTOR signaling, which suppressed the growth and progression of tumor cells.

**Conclusions:**

The results highlight that FeSiNTs/siSPAG5 caused no activation of the innate immune response nor any systemic toxicity, indicating the possible therapeutic utility of FeSiNTs/siSPAG5 to deliver siSPAG5 to treat bladder cancer.

**Graphic abstract:**

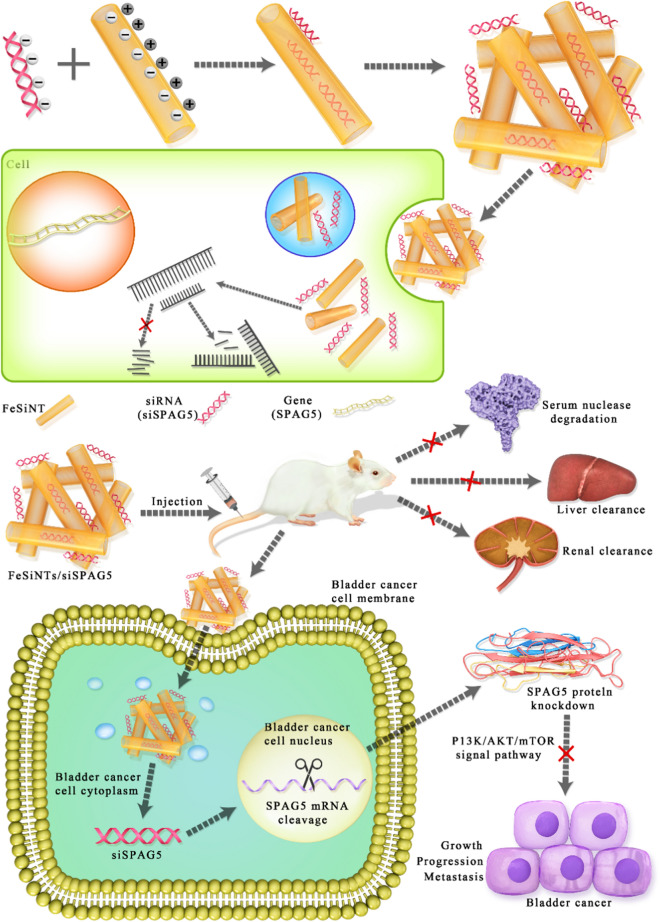

**Supplementary Information:**

The online version contains supplementary material available at 10.1186/s12951-021-00935-z.

## Background

Currently, disrupted regulatory networks and their associated genome aberrations are being investigated by large-scale genomics projects, such as The Cancer Genome Atlas project and the International Cancer Genome Consortium, for their effects in promoting cancer progression [1, 2]. A wide range of therapeutic agents have been developed guided by these large-scale research efforts, which are designed to inhibit cancer-dependent genes and pathways [3–5]. Unfortunately, a large proportion of these therapeutic targets cannot currently be targeted by, or do not respond to, antibodies or small molecule inhibitors [6]. Small interfering RNAs (siRNAs) induce mRNA degradation in a sequence-specific manner, and are used widely to ablate the expression of genes with important functions in cancer cell survival and progression. For instance, siRNA silencing of *p53* [7], *BCL2* (encoding BCL2 apoptosis regulator) [8], and *SPAG5* (encoding sperm associated antigen 5) [9], could regulate human bladder cancer and the growth and progression of other cancer cells. However, in the clinical setting, the use of siRNAs as therapeutics presents several challenges [10]: The administration of naked siRNAs into the human or animal blood stream would result in their degradation by blood-borne nucleases; and the negative charge on siRNAs prohibits them from crossing target cells plasma membranes to achieve gene knockdown. Therefore, an effective siRNA carrier should be developed to establish siRNA therapy.

Naturally aluminosilicate minerals with nanosized structures (e.g., halloysite, kaolinite, and montmorillonite) have been constructed to efficiently deliver siRNAs because of their stable chemical composition, micro-morphology structures, and unique physicochemical properties. Precise control over the main physicochemical features, such as the size, shape, and charge, is essential for the natural aluminosilicate minerals used in the field of bio-medical engineering. Chrysotile (Mg_3_Si_2_O_5_(OH)_4_) is a 1:1 hollowed tubular morphology clay mineral consisting of one tetrahedral SiO_4_ sheet outer surface and one octahedral gibbsite Mg(OH)_3_ sheet inner surface. Despite asbestos long-term exposure could cause chronic pleural diseases, pulmonary fibrosis and lung cancers, which strongly depending on length-diameter ratio and possibly metal content, the synthetic hydrosilicate chrysotile with a length of several hundred nanometers possess little toxic potential compared to natural chrysotile with a length above 10 μm. According to previous literatures, lung cancer and pulmonary fibrosis caused by asbestos long-term exposure with longer than ~ 15 µm and thicker than 0.1 µm, mesotheliomas and pleural plaques caused by asbestos long-term exposure with longer than ~ 4–5 µm and thinner than ~ 0.1 µm. Geoinspired synthetic chrysotile nanotubes have been prepared using hydrothermal synthesis with the composition (Mg, Fe, Co, Ni)_3_Si_2_O_5_(OH)_4_, and specific properties, such as optical, electronic, and magnetic properties, have been achieved. Previous studies suggest that natural minerals could be synthesized and tailored for cancer therapeutic applications [11–13].

Consequently, the present study aimed to use Fe-doped chrysotile nanotubes (FeSiNTs) to deliver an anti-cancer siRNA targeting the *SPAG5* oncogene. *SPAG5* is located at 17q11, a frequently amplified region, and encodes a protein involved in mitotic spindle assembly. Recently, *SPAG5* has been suggested a novel oncogene in various cancers. Studies suggest that *SPAG5* is involved in tumorigenesis and cancer progression. The expression of *SPAG5* is increased in various tumor tissues, such as breast cancer, prostate cancer, lung cancer, hepatocellular carcinoma, gastric cancer, and cervical cancer, and upregulated *SPAG5* is associated with poor prognosis in cancer patients [14–18]. Accordingly, our previously data suggested that *SPAG5* upregulation could be detected frequently in primary bladder cancer tissues and high *SPAG5* expression was identified a novel independent prognostic marker for patient survival [9]. *SPAG5* is associated with tumorigenesis, apoptosis, and the tumor cell cycle in vitro and in vivo [19–21]. Moreover, *SPAG5* knockdown resulted in marked anti-tumor effects in a number of human malignancies [19–21].

In this study, we created Geoinspired synthetic FeSiNTs, consisting of a special nanostructure combining Fe cations occupying octahedral sites and a tubular morphology, which could be used for the encapsulation, sustained release, and intracellular delivery of siRNAs. We used these siRNA-delivering nanoparticles to treat bladder cancer. Moreover, FeSiNTs could efficiently and safely deliver siSPAG5 into bladder cancer cells, where they escaped from endosomes into the cytosol. We showed that siSPAG5 delivered by FeSiNTs nanoparticles could effectively inhibit the migration, invasion, and proliferation of bladder cancer cells.

## Results

### Synthesis and characterization of FeSiNTs

**Fig. 1:**
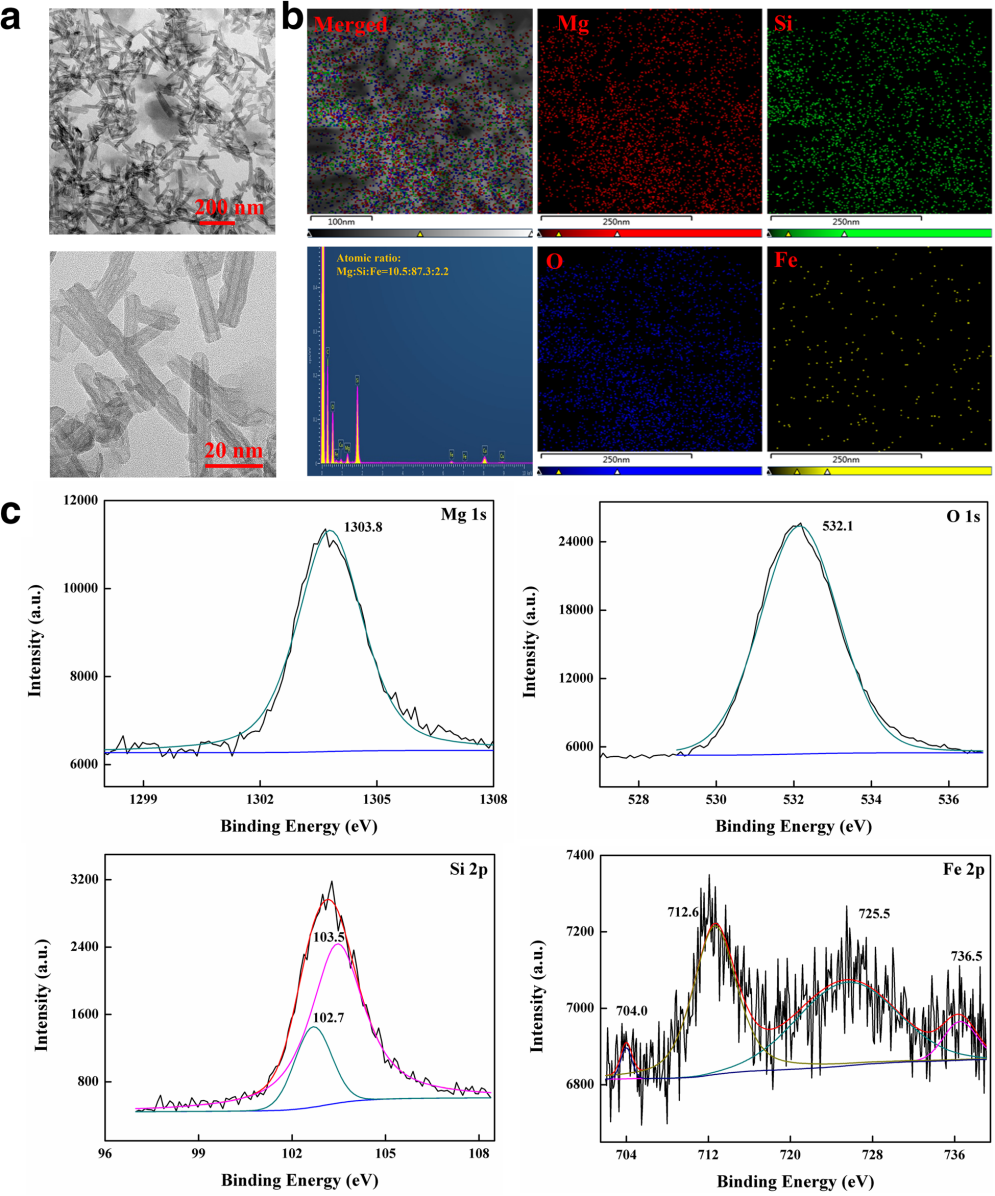
Characterization of the siRNA-loaded FeSiNTs. **A** TEM images and **B** EDS elemental mapping of FeSiNTs. **C** The XPS survey spectrum of Mg 1 s, O 1 s, Si 2p, and Fe 2p spectra of FeSiNTs

The fixed size, morphology, and chemical composition of chrysotile could be used to ensure that the toxicity evaluation is objective, fair, and accurate. FeSiNTs were prepared with an Fe content up to 1.37 wt.% under the different hydrothermal environments. The Fe doping extent of synthesized chrysotile nanotubes using the hydrothermal method reported in literature ranged from 0.29 to 1.37% [22, 23]. The TEM images showed that the FeSiNTs had a hollow tubular morphology similar to those reported previously [24], with a relatively uniform outer diameter (7–8 nm), inner diameter (3–5 nm), and a length of several hundred nanometers (Fig. 1A; Additional file 1: Figure S1, Additional file 2: Figure S2 and Additional file 3: Figure S3A). Fe was homogeneously distributed into the layer structure, as shown by the Energy Dispersive Spectrometer mapping (EDS) maps (Fig. 1B), and indicated that Fe elements were substituted into octahedral sites with an appropriate mass ratio. Comparison of the XRD patterns (Additional file 3: Figure S3B) showed peaks corresponded to magnesite (JCPDS Card No. 08–0479) and lizardite (JCPDS Card No. 50-1625). The N_2_ adsorption isotherms (Additional file 3: Figure S3C) showed the features expected for microporous systems with some mesoporosity, with a Barrett-Joyner-Halenda (BJH) pore diameter of about 7–8 nm, the Brunauer Emmett Teller (BET) specific surface area ranged from 80 to 150 m^2^/g, and the pore volume was approximately 0.45 mL/g. The isoelectric point was approximately 3.5 (Additional file 3: Figure S3D), and most of the zeta potentials were positive between 45.7 mV (pH 2.0) and − 8.42 mV (pH 10.0). The peak fitting program on the XPS spectra (Fig. 1C) showed that the binding energy (BE) value of O 1 s, Mg 1 s, Si 2p in silanol (Si–OH) and silicon-oxygen (Si–O), were located at 532.1 eV, 1303.8 eV, 103.5 eV, and 102.7 eV, respectively. Peaks at 712.6 eV (Fe2p3/2) and 725.5 eV (Fe2p1/2) correspond to γ-Fe_2_O_3_, while the satellites peaks at 704.0 eV and 736.5 eV indicated that Fe_3_O_4_ was contained.

### FeSiNTs siRNA-binding efficiency and cytotoxicity

The RNA-binding ability of FeSiNTs complexes were tested using agarose gels. FeSiNTs could bind siRNAs in a dose-dependent manner (Additional file 4: Figure S4A), as determined by the level of un-complexed (free) siRNA in the agarose gel. At mass ratios of 30:1 or higher, in the mixture of FeSiNTs with siRNA, the siRNA band was almost absent compared with that of the free siRNA lane, which implied that the FeSiNTs complexes had a high binding ability for siRNA.

FeSiNTs cytotoxicity was assessed using the Cell counting kit-8 (CCK-8) assay. Cytotoxicity was assessed using FeSiNTs delivering the NC-siRNA in T24 cells at different concentrations. No obvious toxicity was observed even at the highest concentration of FeSiNTs complexes (200 μg/mL), a level that was 20 times higher than the concentration required for effective transfection (Additional file 4: Figure S4B; Additional file 5: Figure S5). By contrast, Lipofectamine 3000 demonstrated some cytotoxicity at the transfection effective dose. The lack of cytotoxicity suggested that FeSiNTs could be suitable as an siRNA transfer device.

### Release analysis of FeSiNTs/siRNA in medium and serum

The release of FeSiNTs/siRNA in medium and serum was tested by gel electrophoresis. As shown in Additional file 6: Figure S6, the free siRNA was unstable in medium and serum, and the free siRNA bands disappeared after incubation in medium and serum for 16 h and for 8 h, respectively. However, when FeSiNTs/siRNA was incubated with serum and medium for 32 h, the integrity of the FeSiNTs/siRNA band was still evident (Additional file 6: Figure S6).

### FeSiNTs transfection efficiency

**Fig. 2:**
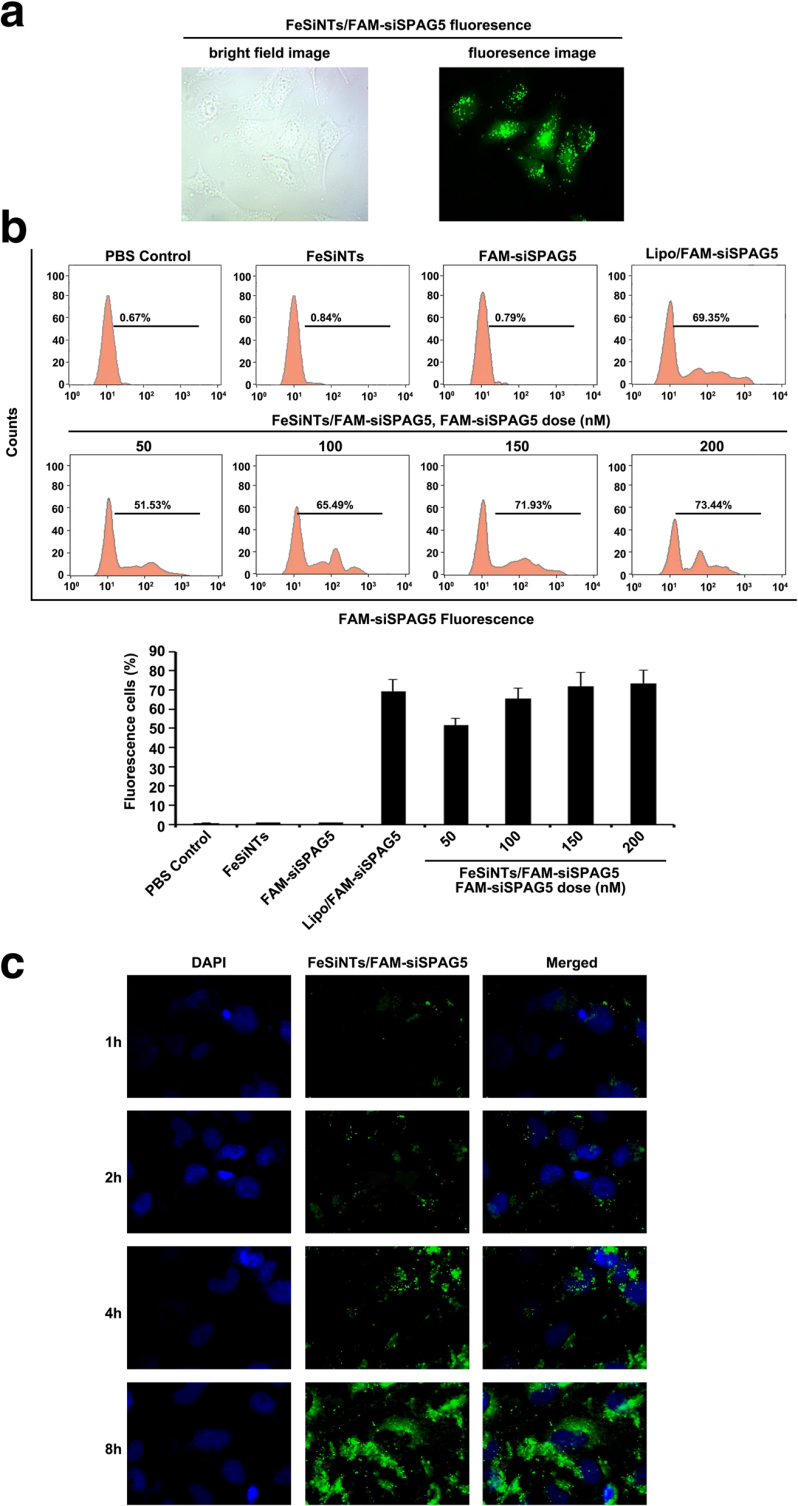
The transfection efficiency and distribution of FeSiNTs-delivered siSPAG5 in T24 bladder cancer cells. **A** Bright field and fluorescence microscopy images of FeSiNTs/FAM-siPAG5 fluorescence at 6 h after transfection. **B** Flow cytometry analysis of fluorescent cells: representative histograms (upper panel) and the mean ± SD (lower panel, from three independent experiments). FAM-siSPAG5: fluorescein-labelled small interfering RNA targeting *SPAG5*. **C** Confocal laser scanning microscopy (CLSM) analysis of the distribution of FeSiNTs/FAM-siSPAG5 in T24 bladder cancer. Fluorescein (green) labeled siSPAG5. DAPI (4ʹ,6-diamidino-2-phenylindole; blue) was used to stain cell nuclei

The cellular uptake of FeSiNTs/fluorescein (FAM)-siRNA complexes by T24 cells was determined using inverted fluorescence microscopy. After 6 h of incubation, FAM-siRNA could be observed inside T24 cells, demonstrating FeSiNTs internalization (Fig. 2A).

To determine how the formulations’ composition influenced siRNA internalization, FeSiNTs/FAM-siRNAs at various concentrations were incubated with T24 cells for 4 h and then flow cytometry was performed after quenching the extracellular fluorescence. The percentage of fluorescent cells (containing FeSiNTs/FAM-siRNA complexes) increased in a FeSiNTs/FAM-siRNA dose-dependent manner (Fig. 2B). Moreover, at an FeSiNTs/FAM-siRNA dose of 150 nM, the cells showed an approximately similar efficiency of transfection compared with that of Lipofectamine 3000/FAM-siRNA complexes. The efficiency of transfection changed little at doses of siRNA up to 200 nM. Thus, the optimal dose of siRNA delivered by FeSiNTs transfection of T24 cells was determined as 150 nM. Importantly, at a dose inducing transfection efficiency similar to that Lipofectamine 3000, no obvious cytotoxicity of FeSiNTs was observed.

### Cellular uptake and endosomal escape of FeSiNTs-siRNA

To achieve efficient gene silencing using siRNAs, high levels of cell uptake and successful release of the siRNA into the cytoplasm [25]. Thus, T24 cells incubated with FeSiNTs/FAM-siRNA for various times were examined for the nanoparticle locations in cells whose nuclei were counterstained with 4ʹ, 6ʹ-diamidino-2-phenylindole (DAPI). At the beginning, the FAM-siRNA fluorescence was distributed a punctate manner in the cytoplasm and nuclear periphery. At 4 and 8 h, the green fluorescence was significantly increased (Fig. 2C).

To confirm the lysosomal escape of FeSiNTs-siRNA, LysoTracker was used to stain T24 cells, followed by confocal fluorescence microscopy (Additional file 7: Figure S7). After incubation for 1 h, FeSiNTs-siRNA (green) and LysoTracker (red) fluorescence were co-localized in the transfected cells (Additional file 7: Figure S7), demonstrating the lysosomal location of the FeSiNTs-siRNA. At 4 h, the green (FeSiNTs-siRNA) and red (LysoTracker) fluorescence had separated (Additional file 7: Figure S7), indicating the lysosomal location of the FeSiNTs-siRNA. At 4 h, the green fluorescence (FeSiNTs-siRNA) had separated from the red fluorescence (LysoTracker; Additional file 7: Figure S7), indicating lysosomal escape of the FeSiNTs-siRNA into the cytoplasm. Therefore, we were confident that the siRNA escaped from the lysosomes as FeSiNTs-siRNA into the cytoplasm where it could perform siRNA-mediated gene silencing.

### SPAG5 knockdown by FeSiNTs-siRNA complex in bladder cancer cells

**Fig. 3:**
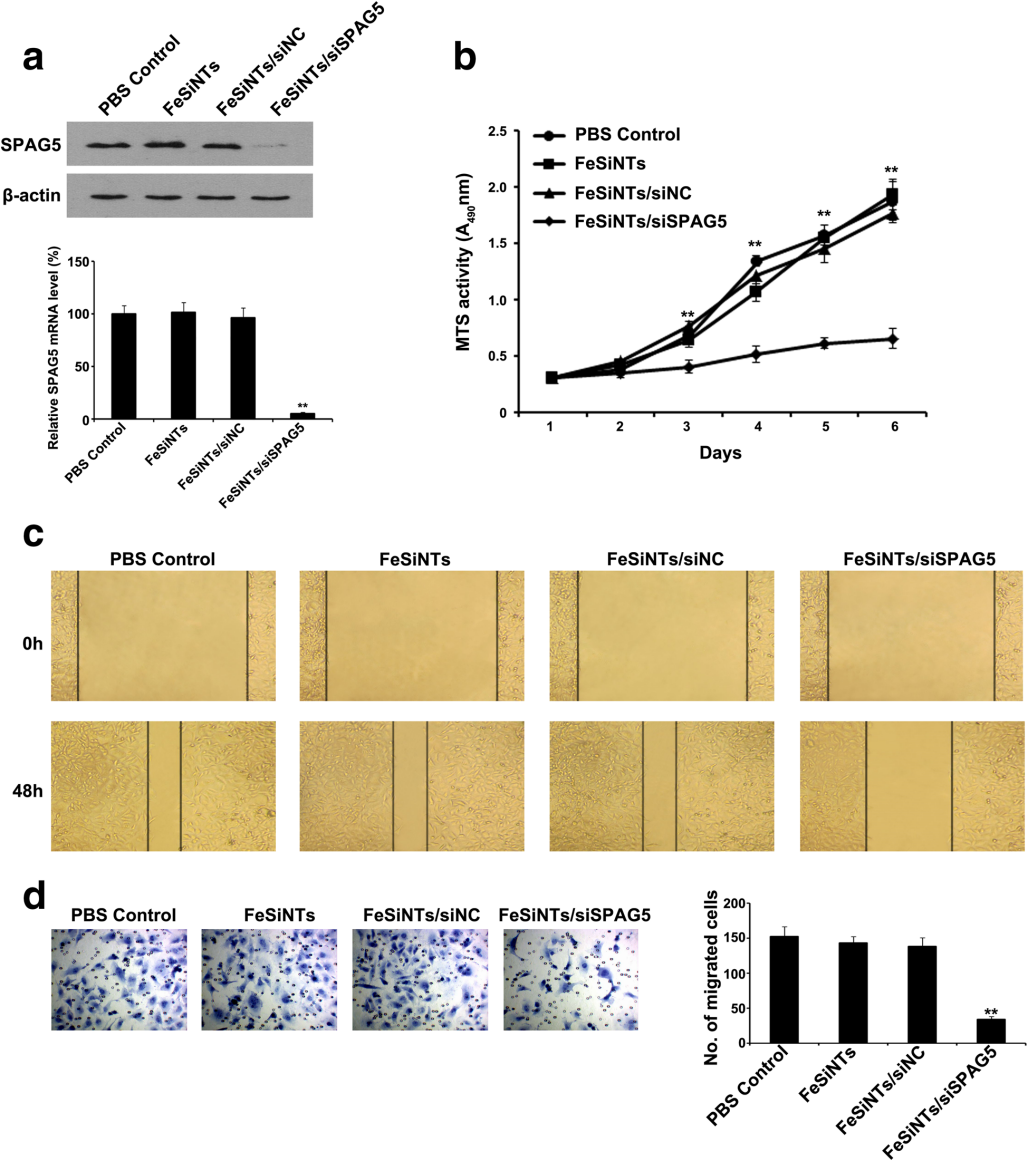
In vitro knockdown of *SPAG5* by FeSiNTs-small interfering RNA treatment inhibits cell proliferation, migration, and invasion of T24 cells. **A** SPAG5 protein and mRNA levels in T24 cells upon transfection with PBS, FeSiNTs, FeSiNTs/siNC, and FeSiNTs/siSPAG5 for 48 h, analyzed by western blotting and qRT-PCR. **B** Effects of *SPAG5* silencing by FeSiNTs/siSPAG5 on T24 proliferation, detected using MTT assays. **C** The migration ability of T24 cells was assessed using an in vitro scratch wound-healing assay. Images were taken 0 and 48 h after wounding of the cell monolayers. **D** The invasiveness of the T24 cells was evaluated using a Transwell assay, and images were captured at 48 h after incubation of the cells in a Matrigel-precoated Transwell chamber (original magnification, ×200). **P < 0.01

Next, we investigated the *SPAG5* gene silencing effect of the FeSiNTs-siRNA complexes in T24 cells. Treatment with FeSiNTs-siRNA, at 150 nM siRNA equivalent, resulted in 95% reduction of *SPAG5* mRNA expression compared with that in the untreated control (Fig. 3A). Correspondingly, in T24 cells treated with FeSiNTs-siRNA, the level of the SPAG5 protein was reduced markedly compared with that in the untreated control (Fig. 3A). Thus, FeSiNTs-siRNA silenced *SPAG5* expression in a very sequence-specific manner. The FeSiNTs protected the siRNAs from degradation and promoted their uptake by cells. The FeSiNTs-siRNA also demonstrated lysosomal escape to exert siRNA-mediated gene silencing.

### FeSiNTs/siSPAG5 regulates the cell cycle, apoptosis, colony formation, and proliferation of bladder cancer cells

We tested the effect of FeSiNTs/siSPAG5 complex-induced *SPAG5* silencing on T24 cell growth using the CCK-8 and EdU assay. T24 cell proliferation gradually decreased after treatment with FeSiNTs/siSPAG5 compared with cells treated with PBS, FeSiNTs, and FeSiNTs/siNC (Fig. 3B; Additional file 8: Figure S8). Among the four treatments, the colony forming ability of the FeSiNTs/siSPAG5-treated group was the lowest (Additional file 9: Figure S9). Thus, silencing of *SPAG5* via treatment with FeSiNTs/siSPAG5 specifically reduced T24 cell proliferation in vitro.

To confirm that apoptosis was involved in the response of T24 cells to FeSiNTs/siSPAG5, the cells were analyzed using flow cytometry with annexin V and PI staining. At 48 h after FeSiNTs/siSPAG5 transfection, the rate of cell apoptosis (annexin-V+/PI− and annexin-V+/PI+) was higher compared with that in cells treated with PBS, FeSiNTs, or FeSiNTs/siNC. The inhibition of *SPAG5* by FeSiNTs/siSPAG5 increased the proportion of apoptotic cells significantly (32.91%) compared with that of the cells treated with PBS, FeSiNTs, and FeSiNTs/siNC (Additional file 10: Figure S10). Flow cytometry assessment of the T24 cell cycle under various treatments was performed. At 2 days post-transfection, there was a reduced proportion of S-phase FeSiNTs/siSPAG5-treated T24 cells compared with that of the controls (Additional file 11: Figure S11). At the same time, the PI values for the PBS, FeSiNTs, FeSiNTs/siNC-treated, and FeSiNTs/siSPAG5-treated cells were 0.52, 0.51, 0.50, and 0.39, respectively (Additional file 11: Figure S11). Collectively, the results suggested that FeSiNTs/siSPAG5-mediated *SPAG5* silencing had a marked anti-tumor effect by decreasing proliferation, and inducing cancer cell apoptosis and cell cycle arrest in vitro.

### FeSiNTs/siSPAG5 regulates migration and invasion of bladder cancer cells

To further evaluate the effect of FeSiNTs-SPAG5-mediated silencing of *SPAG5* on T24 cell behavior, we performed a wound-healing assay in transformed T24 cells. The migration of FeSiNTs/siSPAG5-transfected T24 cells was significantly reduced in a time-dependent manner. By contrast, the controls showed no such reduction (Fig. 3C). Next, Matrigel invasion assays were performed. T24 cells transfected with FeSiNTs/siSPAG5 showed reduced invasive behavior compared with that of the control groups (Fig. 3D). Thus, FeSiNTs/siSPAG5-mediated SPAG5 silencing exerted its anti-tumor effect by reducing the migration and invasiveness of bladder cancer cells in vitro.

### The biodistribution of FeSiNTs/FAM-SiSPAG5 in mice when delivered via intratumoral injection

**Fig. 4:**
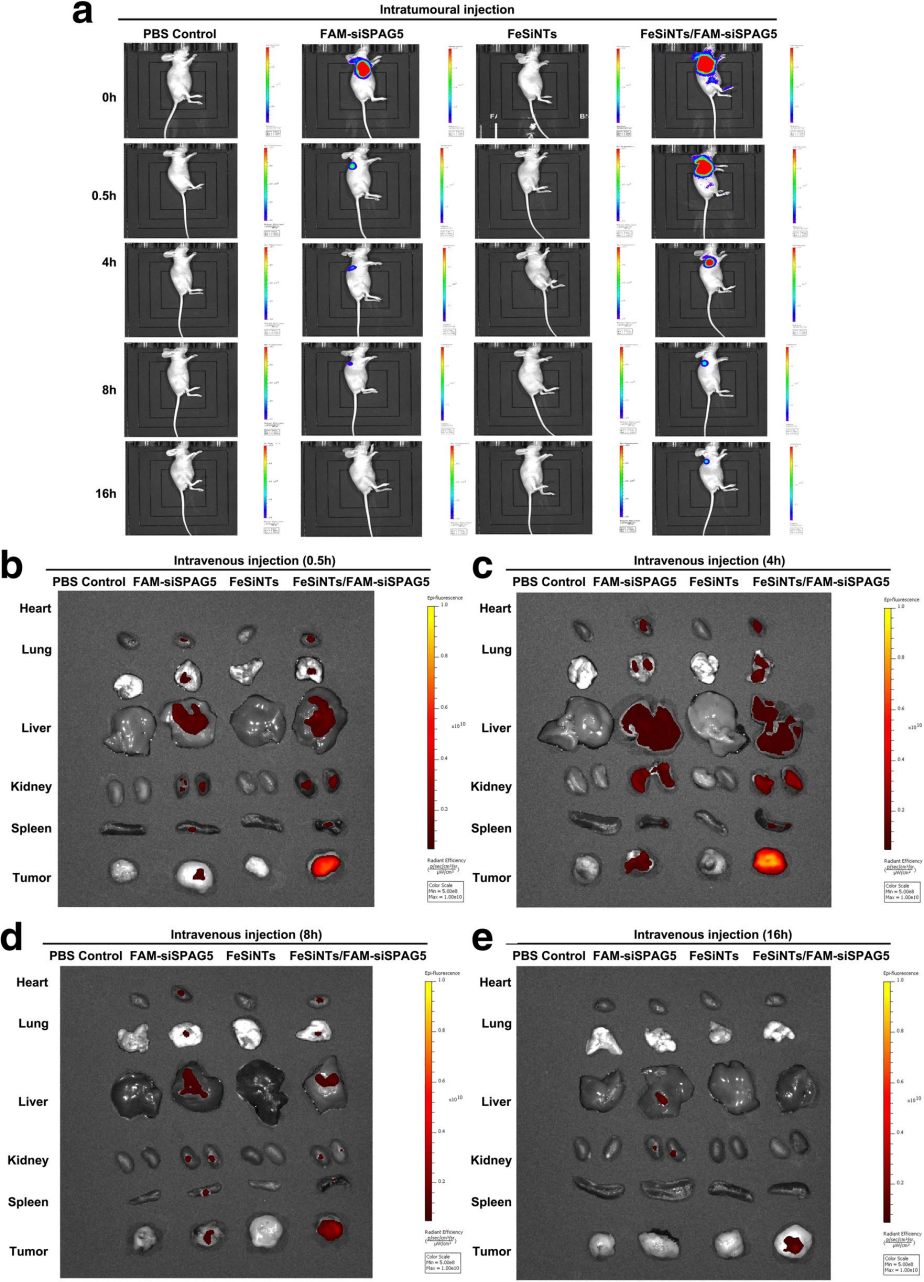
In vivo biodistribution of FeSiNTs/FAM-siSPAG5 monitored using in vivo imaging. **A** In vivo imaging of T24 xenograft-bearing mice after intratumoural injection of FeSiNTs/FAM-siSPAG5, free FAM-siSPAG5, FeSiNTs, or PBS (n = 5 mice in each group). **B**–**E** In vivo imaging of T24 xenograft-bearing mice 0.5 h (**B**), 4 h (**C**), 8 h (**D**), and 16 h (**E**) after tail vein injection of FeSiNTs/FAM-siSPAG5, free FAM-siSPAG5, FeSiNTs, or PBS (n = 5 mice in each group)

Systemic delivery of siRNAs is associated with many adverse effects, which could be reduced by localized delivery (i.e., direct intratumoral delivery) of siRNA. Local administration via direct intratumoral injection of siRNA complexes has been achieved in mouse xenograft models. For example, polyethyleneimine (PEI)-siRNAs could inhibit glioblastoma xenograph tumor growth after intratumoral injection [26]. Intravesicular instillation of chemotherapeutic drugs (e.g., Calmette-Guérin, pirarubicin, and gemcitabine) is accepted widely as a treatment strategy to prevent the postsurgical recurrence of superficial bladder cancer, which avoids the induction of serious adverse events associated with systemic administration. Therefore, we investigated if direct intratumoral injection into a mouse bladder cancer model would result in even siRNA distribution throughout the tumor. FeSiNTs-siSPAG5 was injected intratumorally into mouse subcutaneous xenografts, and in vivo Imaging Technology was utilized to measure the distribution of FAM-siSPAG5. FAM-siSPAG5, FeSiNTs, and PBS were set as controls. At 0.5 h after injection, a stronger fluorescence intensity of FeSiNTs/FAM-siSPAG5 in the tumor tissue compared with that from tissue injected with FAM-siSPAG5 was observed (Fig. 4A). In addition, in the FeSiNTs/FAM-siSPAG5 treated group, the fluorescence distribution area was significantly larger than then that for the naked FAM-siSPAG5 treatment, which demonstrated the excellent tumor tissue penetration ability of FeSiNTs carrying FAM-siSPAG5 and its subsequent distribution over a large tumor area. At 16 h post-injection, the tumor site still showed FeSiNTs/FAM-siSPAG5-related fluorescence, whereas tumors injected with naked FAM-siSPAG5, FeSiNTs, or PBS showed no visible fluorescence. We speculated that the packaging of FAM-siSPAG5 into FeSiNTs inhibited nonspecific adsorption proteins and aggregation of nanoparticles in tumor tissues; therefore, compared with naked FAM-siSPAG5, FeSiNTs/FAM-siSPAG5 accumulated at the tumor site for a longer period.

### FeSiNTs/FAM-siSPAG5 biodistribution in mice via tail vein injection

We next determined whether FeSiNTs nanoparticle-coated siSPAG5 could reach the tumor site by passing through the blood stream, and if they were stable in circulation. PBS, free FAM-siSPAG5, FeSiNTs, or FeSiNTs/FAM-siSPAG5 were injected into mice’s tail veins. The biodistribution of the complexes was determined using Xenogen IVIS Lumina system in mice at 0.5, 4, 8, and 16 h after injection (Fig. 4B–E). It was shown that the fluorescence intensities in bladder tumor tissues collected from the FeSiNTs/FAM-siSPAG5 treated mice were significantly higher than those from the FAM-siSPAG5 treated mice after intravenous injection (Fig. 4B–E). This result suggested that in the absence of the nanoparticle coating, the siRNA was cleared rapidly in vivo via the blood stream. The data implied that in circulation, FeSiNTs/FAM-siSPAG5 were stable, and were delivered efficiently to tumors after tail vein injection.

### Antitumour efficiency

Next, we used three bladder tumor models to investigate the effects of gene knockdown and the antitumor activities of the FeSiNTs-siSPAG5 complexes.

### Gene knockdown and antitumor effect in the subcutaneous model

**Fig. 5:**
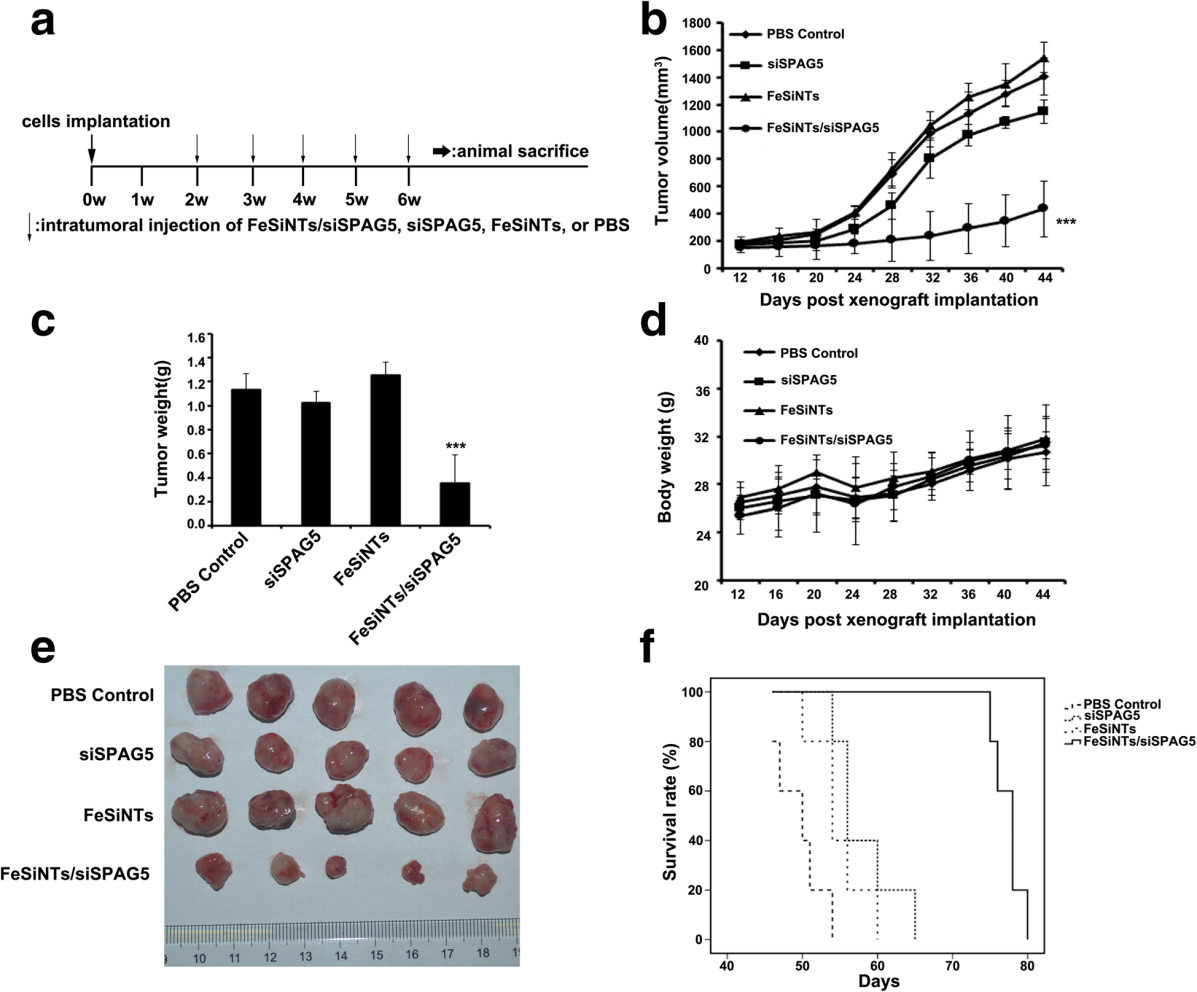
Anticancer activity of FeSiNTs/siSPAG5 in a T24 subcutaneous bladder cancer mouse model. **A** Intratumoral injection of each sample was performed every week in a subcutaneous xenograft model. **B** Tumor volumes, **C** tumor Weight, and **D** the average body weight for 44 days were monitored every 4 days. The tumor volumes were calculated using *V* = (length) × (width) ^2^/2 (mean ± SD, n = 5 mice per group). **E** Representative images of T24 tumor xenografts from the five different mice at day 44. **F** Kaplan–Meier survival plot of tumor-containing mice; dotted lines represent dose administration time points. ***P < 0.001

Subcutaneous tumors were formed by injecting T24 cells into right flank of Balb/c nude mice. FeSiNTs-siSPAG5 complexes, at an siRNA dose of 20 μg per injection, were injected intratumorally weekly for five times in total. Various controls were also injected into separate groups of mice (Fig. 5A). Injection of FeSiNTs-siSPAG5 resulted in the most significant tumor suppression, whereas injection of free siSPAG5 caused limited tumor inhibition (Fig. 5E). Compared with that of the PBS control, the tumor volume for mice treated with FeSiNTs-siSPAG5 was reduced by 69% and was reduced by 19% after free siRNA injection (Fig. 5B). In contrast, tumor growth was not affected by injection of FeSiNTs (Fig. 5B, E). Among all the groups, the FeSiNTs-siSPAG5 group had the smallest tumor sizes and the lowest tumor weights (Fig. 5B, C). Every 4 days, the mice’s body weights were monitored, which revealed no gross toxicity among the groups (Fig. 5D). The mice in the FeSiNTs-siSPAG5 group displayed the longest survival compared with that of the other groups according to Kaplan–Meier survival analysis (Fig. 5F).

We next evaluated the depletion of the SPAG5 protein level in the tumors induced by the FeSiNTs-siSPAG5 complexes. The FeSiNTs-siSPAG5 showed a greater inhibitory effect on SPAG5 protein levels compared with that of the PBS control (Additional file 12: Figure S12). Moreover, immunohistochemistry (IHC) analysis should that the only the FeSiNTs-siSPAG5-treated xenografts showed decreased SPAG5 protein levels (Additional file 13: Figure S13). Subsequently, to determine whether depletion of SPAG5 inhibited tumor cell proliferation, the levels of the proliferation-related protein Ki67 were determined using IHC. Injection of FeSiNTs-siSPAG5 decreased the Ki67 levels (Additional file 13: Figure S13). A TUNEL assay was then used to determine tumor cell apoptosis. Significant numbers of apoptotic cells were observed in tumors injected with FeSiNTs/siSPAG5 (Additional file 13: Figure S13). Thus, *SPAG5* silencing by FeSiNTs-siSPAG5 resulted from increased cellular uptake of FeSiNTs-siSPAG5 complexes compared with that of the free siRNA, and the nanoparticle-delivered siSPAG5 caused RNA interference (RNAi)-mediated gene silencing [27].

### Antitumour effect in in-situ bladder cancer model

**Fig. 6:**
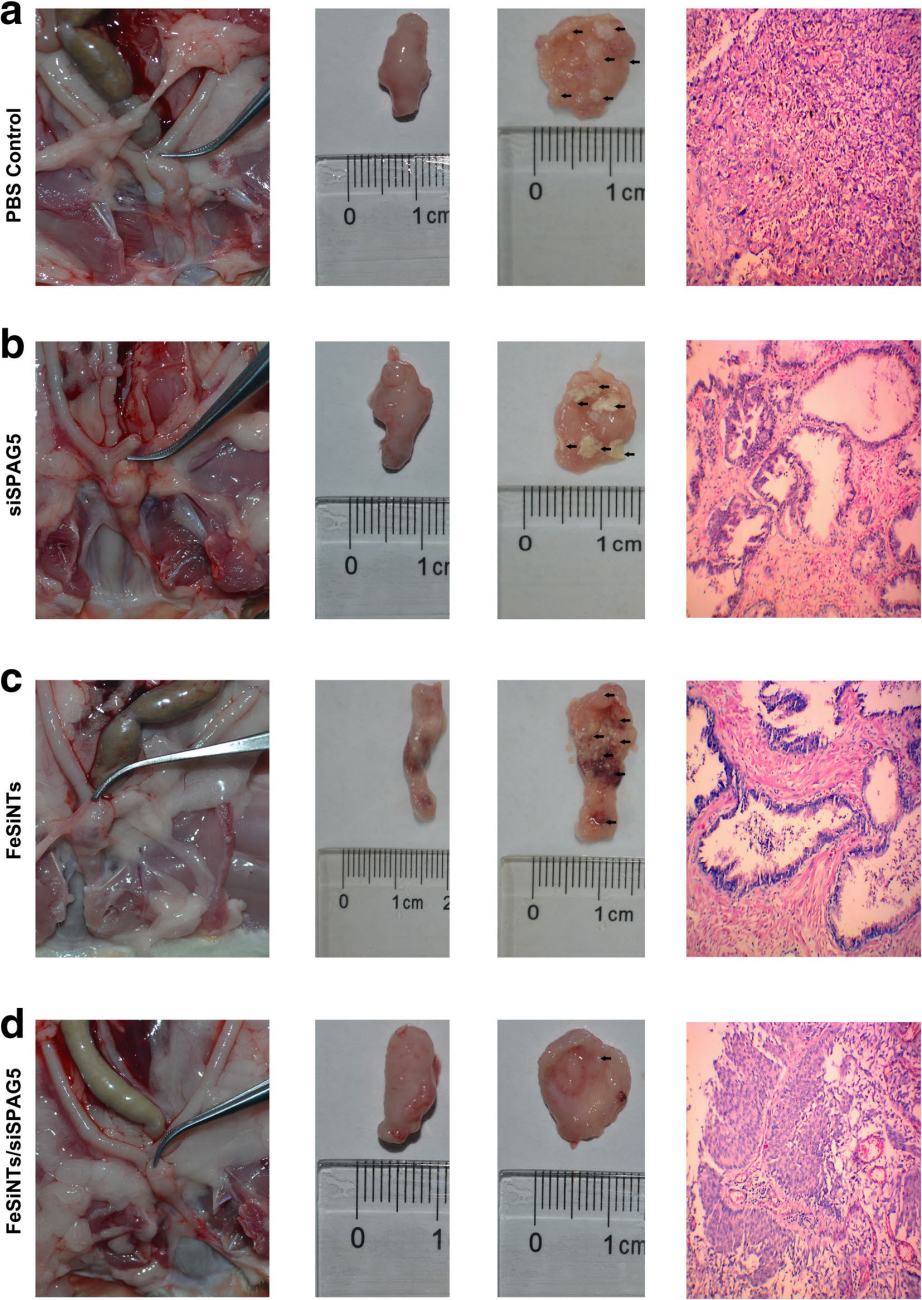
Anticancer activity of FeSiNTs/siSPAG5 in a T24 in-situ bladder cancer mouse model. **A** Representative excised bladders and H&E stained tissue slices (muscle invasive bladder cancer: ≥ stage pT2) treated with PBS. **B** Representative excised bladders and H&E stained tissue slices (muscle invasive bladder cancer: ≥ stage pT2) treated with siSPAG5. **C** Representative excised bladders and H&E stained tissue slices (muscle invasive bladder cancer: ≥ stage pT2) treated with FeSiNTs. **D** Representative excised bladders and H&E stained tissue slices (noninvasive papillary carcinoma: stage pTa) treated with FeSiNTs/siSPAG5

On od the main therapies used to treat bladder cancer is urinary bladder instillation chemotherapy. Therefore, we further investigated the clinical significance of the FeSiNTs-siSPAG5 complexes by determining whether they have a tumor suppressive effect in an in situ model of bladder cancer. Among the treatment groups, the number of the rat’s bladder lesions differed (Additional file 14: Table S1). Significant differences in the histopathological changes were observed among the FeSiNTs-siSPAG5, FeSiNTs, and free SPAG5 treatment groups (*P* < 0.05). Furthermore, treatment with FeSiNTs-siSPAG5 resulted in a lower tumor stage in most of the bladder cancers examined (stage pTa/T1), which contrasted with that in the other groups, in which most of tumors were at a stage higher than pT2. This suggested that the FeSiNTs-siSPAG5 treatment had a markedly better therapeutic effect than PBS treatment. This conclusion was confirmed by histological examination of excised bladders and H&E staining of tissue slices (Fig. 6).

### Antitumour effect in the tail vein injection lung metastasis model

**Fig. 7:**
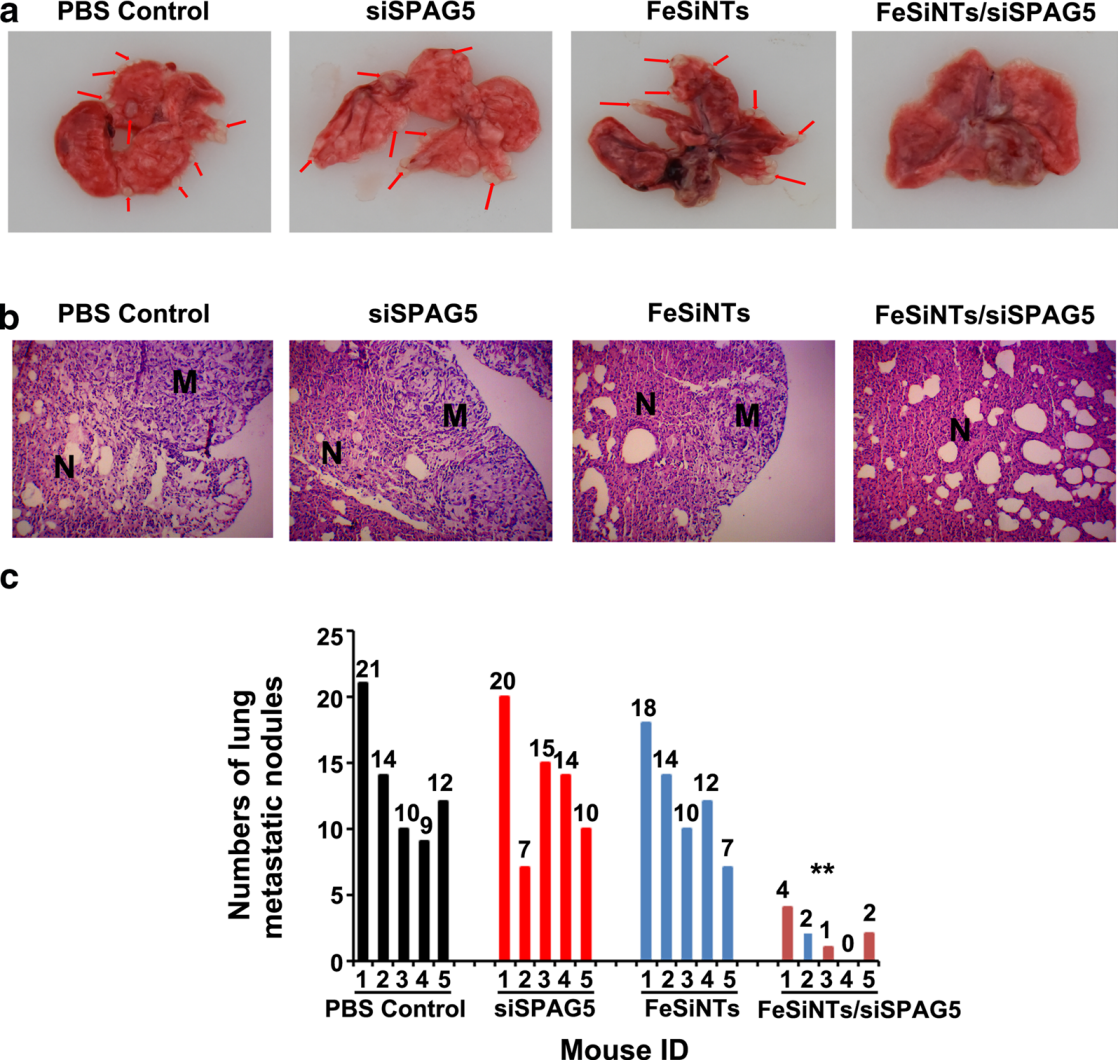
Anticancer activity of FeSiNTs/siSPAG5 in a T24 tail vein injection lung metastasis bladder cancer mouse model. **A** Representative images of lungs and metastatic nodules (indicated by arrows) collected from T24 tumor-bearing mice receiving different treatments. **B** Representative pictures of H&E-stained sections of lungs collected from T24 tumor-bearing mice receiving different treatments. Scale bars represent 200 μm. N: normal lung; M: lung metastatic tumor. **C** The number of metastatic nodules formed in the lungs of BALB/c nude mice is summarized for each group tested. (n = 5). **P < 0.01

We demonstrated highly efficient accumulation of intact FeSiNTs-siSPAG5 in tumors; therefore, the tail vein injection lung metastasis model was used to evaluate the therapeutic efficacy of FeSiNTs-siSPAG5. Pulmonary metastatic nodules were enumerated in the excised lungs from each group of mice. In contrast to those in the PBS and other control groups, almost no macroscopic tumor metastases were seen in the lungs of the FeSiNTs-siSPAG5 group (Fig. 7A, B). The levels of pulmonary metastatic nodules were reduced by 81.8% in the FeSiNTs-siSPAG5 group relative to that in the PBS group (Fig. 7C). Notably, the proportion of metastasis inhibition in the FeSiNTs-siSPAG5 group was significantly larger compared with that in the other groups (*P* < 0.01). The rapid proliferation and growth of tumor cells can promote the occurrence of tumor metastasis and invasion [28], thus the substantial antitumor effect of FeSiNTs-siSPAG5 probably accounts for its demonstrable anti-metastasis efficacy. Thus, the in vivo findings showed that compared with those of the other treatments, the FeSiNTs-siSPAG5 complexes displayed markedly enhanced antitumor efficiency and a substantial antimetastatic effect.

### FeSiNTs-siSPAG5’s in vivo toxicity

We next investigated the in vivo toxicity of the FeSiNTs-siSPAG5 complexes. H&E staining of kidney, lung, spleen liver, and heart sections revealed no obvious histological differences between the FeSiNTs-siSPAG5-treated and the control-treated mice (Additional file 15: Figure S14). These results indicated that treatment with the FeSiNTs-siSPAG5 complexes caused minimal organ toxicity. In the FeSiNTs-siSPAG5 group, the kidney and liver functional parameters were within the normal range (Additional file 16: Table S2). By contrast, in the free siRNA group, the alanine amino transferase (ALT) and aspartate amino transferase (AST; Additional file 16: Table S2) levels were relatively high, suggesting that the free siRNA caused hepatic dysfunction [29]. In addition, treatment with FeSiNTs-siSPAG5 at therapeutic doses resulted in no obvious immune responses, as indicted by the near-control levels of cytokines, such as interleukin-6 (IL-6) and tumor necrosis factor-α (TNF-α), in the FeSiNTs-siSPAG5 group (Additional file 17: Figure S15). By contrast, in the free siRNA group, increased production of IL-6 and TNF-α was observed (Additional file 17: Figure S15) [30]. Taken together, the results demonstrated the high in vivo safety and low toxicity of the FeSiNTs-siSPAG5 complexes.

### PI3K/AKT/mTOR signaling pathway members’ expression in tumor tissues

Next, we performed a preliminary analysis of the downstream signaling pathway by which SPAG5 regulates bladder cancer cell growth and progression. SPAG5 acts as a prognostic indicator in hepatocellular carcinoma and as an oncogene, mediated by the PI3K/AKT pathway [21]. Consistently, during SPAG5 taxol treatment for cervical cancer, SPAG5 was observed to regulate mTOR activity [18]. In addition, in response to oxidative stress, high SPAG5 production is associated with mTOR signaling, which protects cells from apoptosis [31]. Previously, we noted that in bladder cancer, SPAG5 is involved the AKT/mTOR pathway [9]. Consequently, after the animals were sacrificed, the tumors were examined using western blotting and IHC. Consistent with the reduced SPAG5 protein levels induced by FeSiNTs-siSPAG5, western blotting showed significantly decreased expression of PI3K/AKT/mTOR signaling pathway members in the tumor mass (Additional file 12: Figure S12). However, the PI3K, AKT, and mTOR protein levels did not change significantly after treatment with FeSiNTs or free siSPAG5 compared with those in the PBS control. IHC staining of subcutaneous tumor tissue was consistent with the results of western blotting (Additional file 18: Figure S16). Thus, SPAG5 might regulate bladder cancer proliferation and progression via the downstream PI3K/AKT/mTOR signaling pathway.

## Discussion

**Fig. 8:**
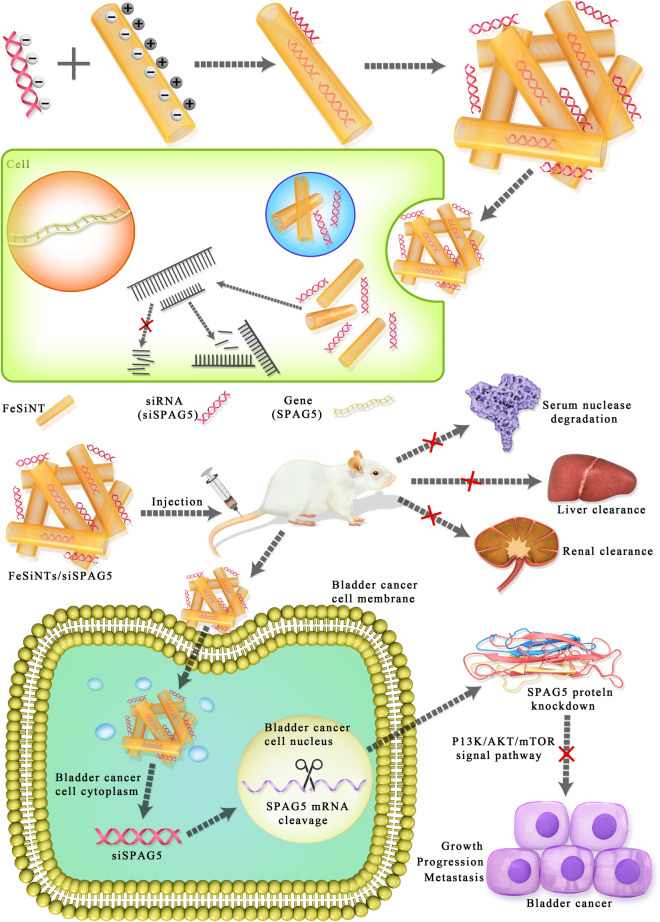
Illustration of the formation of FeSiNTs/siSPAG5 nanomedicine and active targeting, and combinational RNAi therapy in bladder cancer. The FeSiNTs/siSPAG5 complex protects the *SPAG5* siRNA from serum degradation by nucleases and clearance through the kidneys, and promotes *SPAG5* siRNA accumulation in tumor cells, thereby silencing *SPAG5* in bladder tumors. Downregulated *SPAG5* expression inhibits bladder cancer growth and progression

The present study reported the synthesis of an FeSiNTs/SPAG5 siRNA nanoformulation and demonstrated that this carrier system is highly effective to deliver siRNAs into tumor cells. In vivo, it targeted the protooncogenic target *SPAG5* gene, to effectively inhibit the progression and growth of bladder cancer by reducing SPAG5 levels (Fig. 8). In addition, our data suggested that SPAG5 has an important function in the growth and progression of bladder cancer tumors, and its inhibition by FeSiNTs/SPAG5 siRNA nanotherapeutics could be used successfully to target *SPAG5* or other target genes in vivo*.* FeSiNTs-based oligonucleotide therapeutics could from the basis to develop other targeted therapies.

The application of nanoparticles comprising siRNAs has significant advantages over traditional genetic approaches, such as the capacity to alter the expression levels of several genes without recourse to animal breeding, its flexible experimental design, and its translational potential. Although therapies using siRNAs have shown promise, there are significant barriers to their delivery that prevent their clinical translation. The FeSiNTs nanoparticles developed in the present study were designed to incorporate a method of tumor-specific accumulation to solve the challenges presented by siRNA delivery: Protection of siRNAs, their residence time in circulation, cell uptake, site-specific biodistribution, and escape from the endosomal pathway. SPAG5 promotes cell growth and progression in culture; therefore, we hypothesized that silencing of *SPAG5* would affect the survival of cancer cells [9, 16, 19–21]. Therefore, we demonstrated that FeSiNTs-siSPAG5, which integrates elemental iron with siSPAG5, could increase the efficacy of bladder cancer treatment both in vitro and in vivo. FeSiNTs-siSPAG5 effectively suppressed tumors as follows. Fe-doped chrysotile nanotubes show a hollowed tubular morphology structure, which were similar with chrysotile nanotubes consisting of one tetrahedral SiO_4_ sheet inner surface and one octahedral gibbsite Mg(OH)_3_ sheet outer surface. The negatively charged siSPAG5 could be effectively anchored on the positively charged external surface through electrostatic interaction, but it was hard to package into the negatively charged internal surface due to the surface tension and the like charges repel. As other studies in the literature have reported, vacuum impregnation process have been used to increase the encapsulation of Fe-doped chrysotile nanotubes aggregation. Summary, Fe-doped chrysotile nanotubes show that the mixed-charge nanoparticles covered with positively charged external surface and negatively charged internal surface, could passive transport siSPAG5 in bladder cancer cells while no cytotoxicity towards normal cells. In addition, the siRNA and its nanocarriers were cleared by phagocytes initially, and then accumulated in the kidneys and liver before being cleared from the body. Moreover, siSPAG5 induced markedly increased in vivo antitumor activity, as evidenced by the attenuation of PI3K/AKT/mTOR signaling. The PI3K/AKT/mTOR pathway is a major oncogenic driver that exerts vital functions in cancer growth, survival, and progression, and is frequently activated during carcinogenesis. Our findings suggest that the components of FeSiNTs-siSPAG5 each have specific functions, resulting in multiple regulation mechanisms to treat bladder cancer at different stages of delivery.

Conventional nontargeted small-RNA therapeutics cause side effects. To minimize these side effects, we tested the hypothesis that selective delivery to specific cells could be affected by the materials’ physiochemical properties alone. The developed FeSiNTs nanoparticles could transfect bladder cancer cells efficiently in vitro and in vivo, but had no effect on matched normal cells. Immune activation induced by siRNAs causes transient increase cytokines, whose levels increase, peak, and decrease at various times from 1 to 24 h after the first siRNA treatment [32]. Other nanomaterials delivering siRNAs were observed to increase IL-6 and IFN-α levels at 6 h after injection [33], whereas, in the present research, these cytokines showed no significant change after FeSiNTs nanoparticles treatment. We showed that this strategy was suitable for the discovery of siRNA carriers specific for cancer cells. We demonstrated that this strategy was feasible to increase the on-target activity of the siRNA.

## Conclusions

In summary, we have developed FeSiNTs nanoparticles for siRNA targeted delivery to treat bladder cancer cells that overexpress SPAG5. These nanoparticles can efficiently avoid endosome degradation and deliver the therapeutic siRNA into the cytoplasm rapidly, resulting in significantly inhibited expression of *SPAG5* expression and reduced bladder cancer progression and growth. The developed nanoparticles could potentially be used as an siRNA delivery method for robust bladder-directed therapy. Moreover, FeSiNTs-siSPAG5 was well tolerated, without causing marked dose-dependent or treatment-related toxicity. The significance of the present study lies its demonstration of siSPAG5 selective delivery to bladder tumors. Our previously study also showed that > 50% of cases of bladder express SPAG5 [9], suggesting the potential therapeutic utility of targeting SPAG5. Our findings support the use of siRNAs as therapy for patients with bladder cancer.

## Methods

### Cell cultures and materials

T24, a human bladder tumor cell line, was obtained from the ATCC (Manassas, VA, USA). Dulbecco’s modified Eagle’s medium (DMEM; CellGro from Corning, Corning, NY, USA), containing 10% fetal bovine serum (FBS, Gibco, Grand Island, NY, USA), 100 units/mL penicillin and 100 mg/mL streptomycin was used to culture the T24 cells at 37 °C with 5% CO_2_. Molecular Probes (Eugene, OR, USA) provided DAPI. Dojindo Laboratories (Kumamoto, Japan) provided the CCK-8 and BD PharMingen (Heidelberg, Germany) provided the Annexin V-FITC Apoptosis Detection kit. Genepharma (Shanghai, China) synthesized all the siRNAs. The siRNA targeting *SPAG5* comprised the sense strand 5′-AUCUUAAGGAGAGCCAUGATT-3′ and the antisense strand 5′- UCAUGGCUCUCCUUAAGAUGC-3′. The scrambled negative control (NC)-siRNA comprised the sense strand 5′-UUCUCCGAACGUGUCACGUUU-3′ and the antisense strand 5′-ACGUGACACGUUCGGAGAAUU-3′. The fluorescent (FAM, Cy5)-labeled NC-siRNAs had the same sequence, with the dye was attached to the antisense strand. The PCR primers used to detect SPAG5 (forward: 5′-CTGCCCAGTTAGAGGAGTGC-3′, reverse: 5′-TCTGGGTAAGCTGGCAGAGT-3′) and β-actin (forward: 5′-CATTAAGGAGAAGCTGTGCT-3′, reverse: 5′-GTTGAAGGTAGTTTCGTGGA-3′) were synthesized by Sangon Biotech (Shanghai, China).

### Preparation of FeSiNTs-siRNA and characterization

A gel mixture of SiO_2_, 4MgCO_3_⋅Mg(OH)_2_⋅5H_2_O and FeCl_2_⋅4H_2_O with a Si/Mg + Fe molar ratio = 1.50% was used to synthesize the Fe-doped chrysotile nanotubes. The SiO_2_, 4MgCO_3_⋅Mg(OH)_2_⋅5H_2_O and FeCl_2_⋅4H_2_O concentration are 10 mM, 14.8 mM to 13.9 mM and 0.148 mM to 1.0 mM, respectively. Aqueous NaOH (0.4 M) was used to increase the pH of the mixture to 12–14. Hydrothermal treatment was applied at 210–220 °C. The reaction was allowed to proceed for 1 to 3 days, after which the reaction products were dried at 150 °C for 2 h. Fourier transform infrared spectroscopy (FTIR, Nexus-670), X-ray diffraction (DX-2700, XRD), and Transmission electron microscopy (TEM, JEOL JEM-200CX) were used to characterize the microstructure of the composite material.

SiRNA loading: Thirty microliters of a 3 mg/mL FeSiNTs aqueous solution was mixed with the siRNA solution (12 μL; 1 OD unit of siRNA was dissolved in 125 μL of DEPC water), subjected to vacuum impregnation for 3 h, and then centrifuged at 1000 rpm for 5 min. Gel electrophoresis of the supernatant was used to evaluate the siRNA loading efficiency.

### Gel electrophoresis

The FeSiNTs NPs and siRNA were mixed at mass ratios of 1:1, 10:1, 20:1, 30:1, 50:1, and 100:1, and incubated for 30 min at room temperature. Centrifugation for 5 min at 5000 rpm was used to remove insoluble particles. The free siRNA and the complexes in the supernatant were subjected to electrophoresis through a 3% agarose gel containing ethidium bromide (EtBr, 0.1 μg/mL). A UV transilluminator was used to visualize the bands.

### Release analysis of FeSiNTs/siRNA in medium and serum

The FeSiNTs/siRNA and free siRNA were incubated in medium and serum. Centrifugation for 5 min at 5000 rpm was used to remove insoluble particles. At designated time points, samples were stored at − 20 °C until all samples were collected. Samples were then thawed on ice for electrophoresis.

### In vtro siRNA delivery and the FeSiNTs/FAM-siSPAG5 distribution

In three 24-well plates, T24 cells were seeded at 5 × 10^4^ cells/well and then incubated overnight at 37 °C and 5% CO_2_ until they reached 60–80% confluence. The cells were then transfected using various predetermined formulations. At 6 h after transfection, FAM-siSPAG5 uptake by the cells was assessed using an inverted fluorescence microscope (Olympus, IX71, Tokyo, Japan).

T24 cells (5 × 10^4^) were placed into a 35 mm glass bottom culture dish (MatTek Corporation, Ashland, MA, USA) and cultured for at 37 °C in 5% CO_2_ for 24 h, after which the culture medium was replaced by 500 mL of serum-free DMEM containing a 30:1 mass ratio of FeSiNTs/FAM-siSPAG5 complexes. At 6 h after transfection, cells were washed, fixed, and stained using DAPI. Images were obtained using Olympus FluoView confocal laser scanning microscopy (CLSM) and subjected to analysis using FV10-ASW viewer software (Olympus).

### SiRNA lysosomal escape

To determine the lysosomal escape of siRNAs, T24 cells were seeded in a confocal dish at 1 × 10^5^ cells per well for 24 h. Thereafter, LysoTracker Red (75 nm; Invitrogen, Waltham, MA, USA) was used for cell staining for 1 h. Then, stained cells were cultured with free FAM-siRNA (150 nM FAM-siRNA equivalent) and FeSiNTs-siRNA for 1 h at 37 °C. The treated cells were rinsed and cultured in fresh medium for a further 1–6 h. At predetermined time points, aliquots of cells were removed, fixed using 4% PFA, and observed under a confocal microscope. The LysoTracker Red and FAM-siRNA excitation wavelengths were 550 and 480 nm, respectively.

### Quantitative real-time reverse transcription PCR and western blotting

The TRIzol reagent (Invitrogen) was used to extract total cellular RNA, which was converted to cDNA and then subjected to quantitative real-time PCR (qPCR) using *SPAG5*-specific primers and the 7900 Fast RT–PCR system (Applied Biosystems, Foster City, CA, USA) to assess the mRNA expression of *SPAG5*. The endogenous control gene *ACTB* (encoding beta actin) was used to normalize the expression data.

For western blotting analysis, total proteins were prepared from transfected cells. For each treatment, 50 μg of protein was resolved on a 10% SDS gel and the separated proteins were transferred electrophoretically to a polyvinylidene difluoride membrane, which was incubated overnight at 4 °C with rabbit monoclonal antibodies recognizing SPAG5 (Proteintech, Rosemont, IL, USA; 1:1000), phosphorylated (p)-phosphatidylinositol-4,5-bisphosphate 3-kinase (PI3K) (Abcam, Cambridge, MA, USA; 1:1000), p-protein kinase B (AKT) (Abcam, 1:800), and p-mechanistic target of rapamycin (mTOR) (Cell Signaling Technology, Danvers, MA, USA; 1:1000). Thereafter, the membrane was incubated horseradish peroxidase-linked goat anti-rabbit secondary antibody at 37 °C for 1 h (Invitrogen, 1:2000). The internal control was beta-actin (Invitrogen, 1:5000). The ChemiDoc XRS System (Bio-Rad, Hercules, CA, USA) was used to detect the western blotting signals.

### IHC staining

Protein expression was visualized using a Dako Real Envision Kit (Dako, Carpentaria, CA, USA), following to the manufacturer’s protocol, after staining with primary antibodies: anti-SPAG5 antibodies (Proteintech) at a dilution of 1:100, anti-p-PI3K (Abcam) at 1:250, anti-p-AKT (Abcam) at 1:500, anti-p-mTOR (Cell Signaling Technology) at 1:50, and anti-Ki67 (Abcam) at 1:30. To evaluate SPAG5, Ki67, p-PI3K, p-AKT, and p-mTOR IHC staining, we used a semiquantitative scoring system that assessed the staining intensity and positive areas. The staining index (values, 0–12) was calculated using the positive staining intensity (weak, 1; moderate, 2; strong, 3) combined with the immune-positive cell proportion (0%, 0; < 10%, 1; 10–50%, 2; 51–80%, 3; > 80%, 4).

### Cell proliferation analysis

Cell viability was tested with MTT kit (Sigma) according to the manufacturer’s instruction. For colony formation assay, a certain number of transfected cells were placed in each well of 6-well plates and maintained in proper media containing 10% FBS for 2 weeks, during which the medium was replaced every 4 days. Colonies were then fixed with methanol and stained with 0.1% crystal violet (Sigma) in PBS for 15 min. Colony formation was determined by counting the number of stained colonies. EdU experiments were performed using a EdU Cell Proliferation Assay Kit (Cat.C10310-1, Ruibo, Guangzhou, China) according to the manufacturer’s instructions.

### Cell cycle determination

Flow cytometry incorporating propidium iodide (PI) was utilized for cell cycle analysis. Briefly, at 72 h after transfection, T24 cells were pelleted and washed by centrifugation in ice-cold PBS at 125×*g* for 5 min, and then fixed overnight at − 20 °C in 75% ethanol. Then, the cells were subjected to RNase treatment for 30 min at 37 °C prior to PI staining (Bestbio, Shanghai, China) in dark conditions at 4 °C for 60 min. Flow cytometry (Beckman Coulter; Fullerton, CA, USA) was utilized to determine the cell cycle distribution, following the manufacturer’s protocol.

### Assay for apoptosis

A PI kit and Annexin-V-Fluorescein isothiocyanate (FITC) kit (BD Biosciences, San Jose, CA, USA) were used following the manufacturer’s instructions. Pre-chilled PBS was used to wash the cells (2–3 × 10^5^) twice, which were then suspended in binding buffer (100 µL) and 5 μL of FITC-conjugated annexin-V, and incubated in dark conditions at room temperature for 30 min. Thereafter, PI (100 μL) was added and incubation continued for 5 min, before the addition of Binding Buffer (400 µL). Subsequently, a flow cytometer CANTO™ II (BD Biosciences) was used to examine the cells and the data were examined with the aid of the FlowJo software (Becton Dickinson, Franklin Lakes, NJ, USA).

### In vitro invasion and migration assays

Wound-healing, Transwell migration, and Transwell invasion assays were performed to assess the migration and invasion ability of cells in vitro, according to previously published methods [34]. The Transwell migration assay was carried out similarly to invasion assay, except for the addition of the Matrigel (BD Biosciences) coating. The filters with cells were incubated for 48 h at 37 °C and then removed. The adherent cells on the lower surface were fixed and then stained using crystal violet (0.1%; Beyotime). Five randomly selected fields in each well were photographed and the invaded or migrated cells were enumerated under an inverted microscope (Olympus) at a magnification of 200×. These experiments were carried out in triplicate.

### In vivo fluorescence imaging

To generate the xenograft tumor model, T24 cells (0.1 mL a suspension of 1 × 10^7^ cells) were injected into the right flank of male BALB/c nude mice. The mice were divided randomly into four groups when the tumor volume was 200 to 300 mm^3^. For the in vivo imaging study, 400 μL of FeSiNTs/FAM-siSPAG5, the equivalent of free FAM-siSPAG5, FeSiNTs only, or PBS were injected intratumorally. Then, at 0.5, 4, 8, and 16 h post-injection, the Xenogen IVIS Lumina system (Caliper Life Sciences, Hopkinton, MA, USA) was utilized for mouse scanning, with an exposure time of 1 s per image. Living Imaging software (Bio-Real Sciences, Salzburg, Austria) was then used to analyze the images.

Mice were sacrificed at 0.5, 4, 8, and 16 h post-intravenous tail vein injection to assess the in vivo distribution of the complexes. Tissues including the heart, liver, lung, kidney, spleen, and tumor were removed and examined using the Xenogen IVIS Lumina system with Living Image software.

### Antitumour effects in vivo

To investigate *SPAG5* gene silencing and the antitumor effects of the FeSiNTs/siSPAG5 complexes, we constructed three bladder tumor models: A subcutaneous tumor model, an in-situ bladder cancer model, and a tail vein injection lung metastatic model.

### Tumor suppression effect of FeSiNTs/siSPAG5 in a model of subcutaneous bladder cancer

To study FeSiNTs/siSPAG5’s tumor suppressive effects, a subcutaneous bladder cancer model comprising BALB mice with T24 tumors was constructed as described previously. At a tumor volume reached about 100 mm^3^, the mice were placed randomly into four treatment groups comprising five mice per group. The groups were injected subcutaneously with FeSiNTs/siSPAG5 (20 μg of siSPAG5 per injection, 30:1 mass ratio), an equivalent amount of unloaded FeSiNTs, siSPAG5, or PBS solution, respectively. Injections were repeated once per week for 5 weeks. Meanwhile, every 4 d, the short and the long axial lengths of the tumors were measured and the mice were weighed. Kaplan–Meier survival curves were designed to evaluate mouse survival. The tumor xenografts were excised, weighed, and snap-frozen for cryosectioning, or formalin-fixed for paraffin sectioning. Hematoxylin and eosin (H&E) of tumor histological sections was performed. A TdT-mediated dUTP Nick-End Labeling (TUNEL; Colorimetric TUNEL Apoptosis Assay Kit (Beyotime, Haimen, China)) assay of primary tumor sections assessed tumor apoptosis following the supplier’s protocol. A Dako Real Envision Kit (K5007, Dako) was used to perform IHC staining, utilizing primary antibodies to detect protein expression.

### Tumor suppression effect of FeSiNTs/siSPAG5 instillation in an in-situ bladder cancer model

Female SD rats were anesthetized using ether inhalation, and their bladders were infused using 0.2 ml *N*-methyl-Nnitrosurea (MNU) (10 mg/mL; Sigma, St. Louis, MO, USA,) using a 22-gage angiocatheter one every 14 days five times. After catheterization, the rats remained anesthetized for approximately 45 min to avoid spontaneous micturition [35, 36].

After successful tumor induction (approximately 16 weeks), 60 rats were assigned to 4 groups of 15 rats. Then, the rats were anesthetized and 500 μL of FeSiNTs/siSPAG5, siSPAG5 only, an equal dose of free FeSiNTs, or PBS were instilled into the rats’ bladders. The rats stayed sedated for approximately 45 min after instillation to minimize spontaneous micturition. The treatments were repeated once per week for 5 weeks. At 48 h after therapy termination, the rats were killed humanely. Their bladders were excised, and fixed in 4% paraformaldehyde for 24 h, paraffin-embedded, and subjected to histopathological examination. At the midportion of the bladder, transverse sections were cut, followed by H&E staining.

### Tumor suppression effect of FeSiNTs/siSPAG5 instillation in tail vein injection lung metastatic model

T24 cells (2.5 × 10^6^ cells in 100 µL of cold PBS) were injected into BALB/c-nude mice tail veins. At 4 weeks after injection, siRNA therapy was initiated. The mice were divided randomly into four groups of 5 and treated with 0.3 mg/kg of FeSiNTs/siSPAG5, an equivalent dose of siSPAG5 only, an equivalent dose of free FeSiNTs, or PBS solution once a week for 5 weeks through their tail veins. After treatment for 5 weeks, the mice were euthanized with CO_2_ and lung tissues were removed for histological examination and imaging. We then counted the macroscopic metastatic nodules in each lung.

### In vivo cytotoxicity

For in vivo toxicity assessment of the FeSiNTs/siSPAG5, further mouse groups (n = 5 per group) were injected via their tail vein with 25 μg FeSiNTs/siSPAG5, an equivalent dose of siSPAG5 only, an equivalent dose of free FeSiNTs, or PBS solution. Twenty-four hours later, the animals were sacrificed and their heart, liver, spleen, lungs and kidney were removed and formalin (10%)-fixed. Fixed tissues were embedded in paraffin and H&E stained using standard protocols.

### Biochemical assays

Twenty-four hours post-tail vein injection, blood samples were obtained from the mice and subjected to centrifugation to obtain the serum, which was stored at − 20 °C for further analysis. Modular analytics (Roche, Germany) was used to determine the serum AST, ALT, creatinine (CREA), and blood urea nitrogen (BUN). For the immunotoxicity assay, enzyme-linked immunosorbent assay (ELISA) kits (Abcam) were used to measure serum cytokine levels (interleukin (IL)-1β, IL-6, Iinterferon alpha (IFN-α), and TNF-α).

### Statistics analysis

GraphPad Prism VI software (GraphPad Software, Inc., La Jolla, CA, USA) was utilized for statistical analysis. Student’s t test evaluated the statistical significance between groups and one-way analysis of variance (ANOVA) was used for multiple comparisons. Statistical significance was accepted at *P* < 0.05. Data are shown as the mean ± the standard error of the standard deviation (SD).

## Supplementary Information

**Additional file 1: Figure S1.** Large-scale TEM images of FeSiNTs with different hydrothermal environments.

**Additional file 2: Figure S2.** Partial enlarged TEM images of FeSiNTs with different hydrothermal environments.

**Additional file 3: Figure S3.** (A) DSL data, (B) XRD patterns, (C) Nitrogen adsorption–desorption isotherms, and(D)Zeta-potential of FeSiNTs with different hydrothermal environments.

**Additional file 4: Figure S4.** siRNA-binding efficiency (A) and cytotoxicity (B) of FeSiNTs.

**Additional file 5: Figure S5.** Cytotoxicity of FeSiNTs detected by EdU assays. **P < 0.01.

**Additional file 6: Figure S6.** Relaease analysis of FeSiNTs/siRNA after incubation with medium (A) and serum (B) for different times, as determined by gel electrophoresis.

**Additional file 7: Figure S7.** Lysosomal escape of FeSiNTs/siSPAG5 in T24 cells.

**Additional file 8: Figure S8.** Effects of *SPAG5* silencing by FeSiNTs/siSPAG5 on T24 proliferation, detected using a EdU assay.

**Additional file 9: Figure S9.** Effects of *SPAG5* silencing by FeSiNTs/siSPAG5 on the colony formation ability of T24 cells, tested using a plate colony assay. **P < 0.01.

**Additional file 10: Figure S10.** Annexin V and propidium iodide staining of T24 cells treated with FeSiNTs/siSPAG5 complexes and other formulations, and analysis of apoptosis using flow cytometry. **P < 0.01.

**Additional file 11: Figure S11.** After FeSiNTs-mediated *SPAG5* knockdown in T24 cells, FACS was used to analyze the cell cycle. **P < 0.01.

**Additional file 12: Figure S12.** Tumour tissues analyzed using western blotting from the subcutaneous xenograft model.

**Additional file 13: Figure S13.** SPAG5 and Ki67 immunohistochemistry analysis, and TUNEL staining analysis of the tumors treated with PBS, siSPAG5, FeSiNTs, and FeSiNTs/siSPAG5. **P < 0.01.

**Additional file 14: Table S1.** Tumor suppression effect of different treatment on bladder histopathologic changes in SD rat bladders of different groups.

**Additional file 15: Figure S14.** Biosafety evaluation of FeSiNTs/siSPAG5 in vivo.

**Additional file 16: Table S2.** Biochemical parameters of blood from the mice at 24 h after last injection of various drugs

**Additional file 17: Figure S15.** IFN-α (A), IL-1β (B), IL-6 (C), and TNF-α (D) analysis of mouse blood at 24 h after PBS, siSPAG5, FeSiNTs, and FeSiNTs/siSPAG5 injection.

**Additional file 18: Figure S16.** Immunohistochemistry analysis of members of the PI3K/AKT/mTOR signaling pathway in tumor tissues treated with PBS, siSPAG5, FeSiNTs, and FeSiNTs/siSPAG5. **P < 0.01.

## Data Availability

All data generated or analyzed during this study are included in this published article.
