# Supplementary material for: Fe-doped chrysotile nanotubes containing siRNAs to silence SPAG5 to treat bladder cancer
Source: J Nanobiotechnology. 2021 Jun 23;19:189. doi: 10.1186/s12951-021-00935-z (PMC8220725; doi:10.1186/s12951-021-00935-z)
Supplement: Supplementary file 1 — Additional file 1: Figure S1. Large-scale TEM images of FeSiNTs with different hydrothermal environments. [file 12951_2021_935_MOESM1_ESM.docx]

**Additional information**


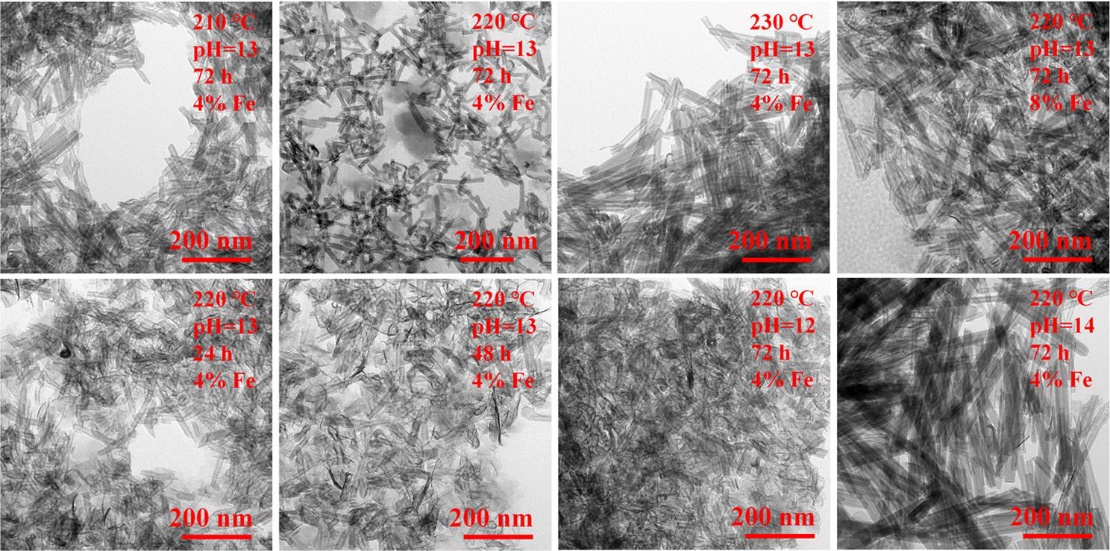


**Additional file 1: Figure S1 Large-scale TEM images of FeSiNTs with different hydrothermal environments.**
