# Supplementary material for: Fe-doped chrysotile nanotubes containing siRNAs to silence SPAG5 to treat bladder cancer
Source: J Nanobiotechnology. 2021 Jun 23;19:189. doi: 10.1186/s12951-021-00935-z (PMC8220725; doi:10.1186/s12951-021-00935-z)
Supplement: Supplementary file 2 — Additional file 2: Figure S2. Partial enlarged TEM images of FeSiNTs with different hydrothermal environments. [file 12951_2021_935_MOESM2_ESM.docx]

**Additional information**


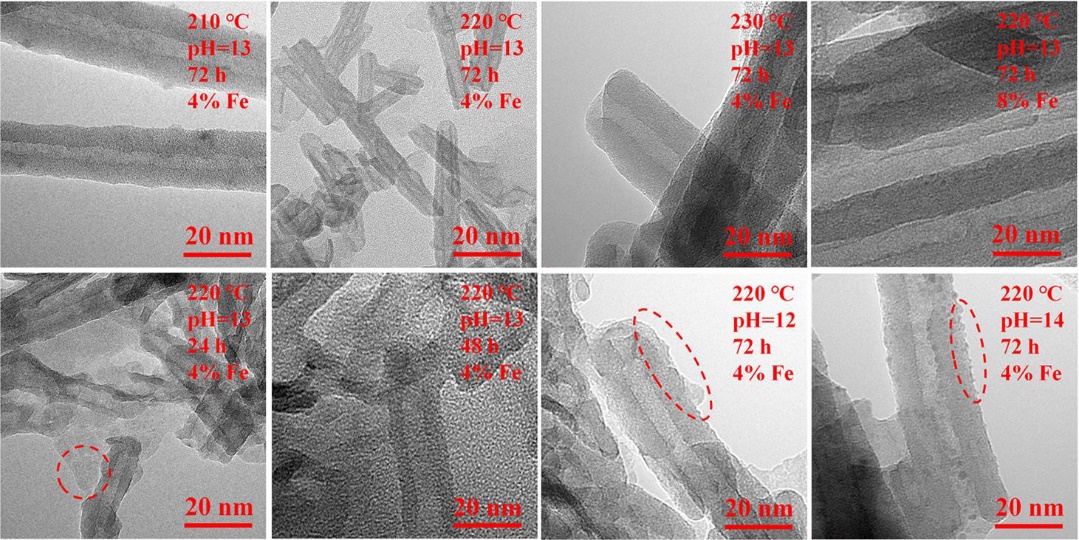


**Additional file 2: Figure S2 Partial enlarged TEM images of FeSiNTs with different hydrothermal environments.**
