# Supplementary material for: Fe-doped chrysotile nanotubes containing siRNAs to silence SPAG5 to treat bladder cancer
Source: J Nanobiotechnology. 2021 Jun 23;19:189. doi: 10.1186/s12951-021-00935-z (PMC8220725; doi:10.1186/s12951-021-00935-z)
Supplement: Supplementary file 3 — Additional file 3: Figure S3. (A) DSL data, (B) XRD patterns, (C) Nitrogen adsorption–desorption isotherms, and(D)Zeta-potential of FeSiNTs with different hydrothermal environments. [file 12951_2021_935_MOESM3_ESM.docx]

**Additional information**

**
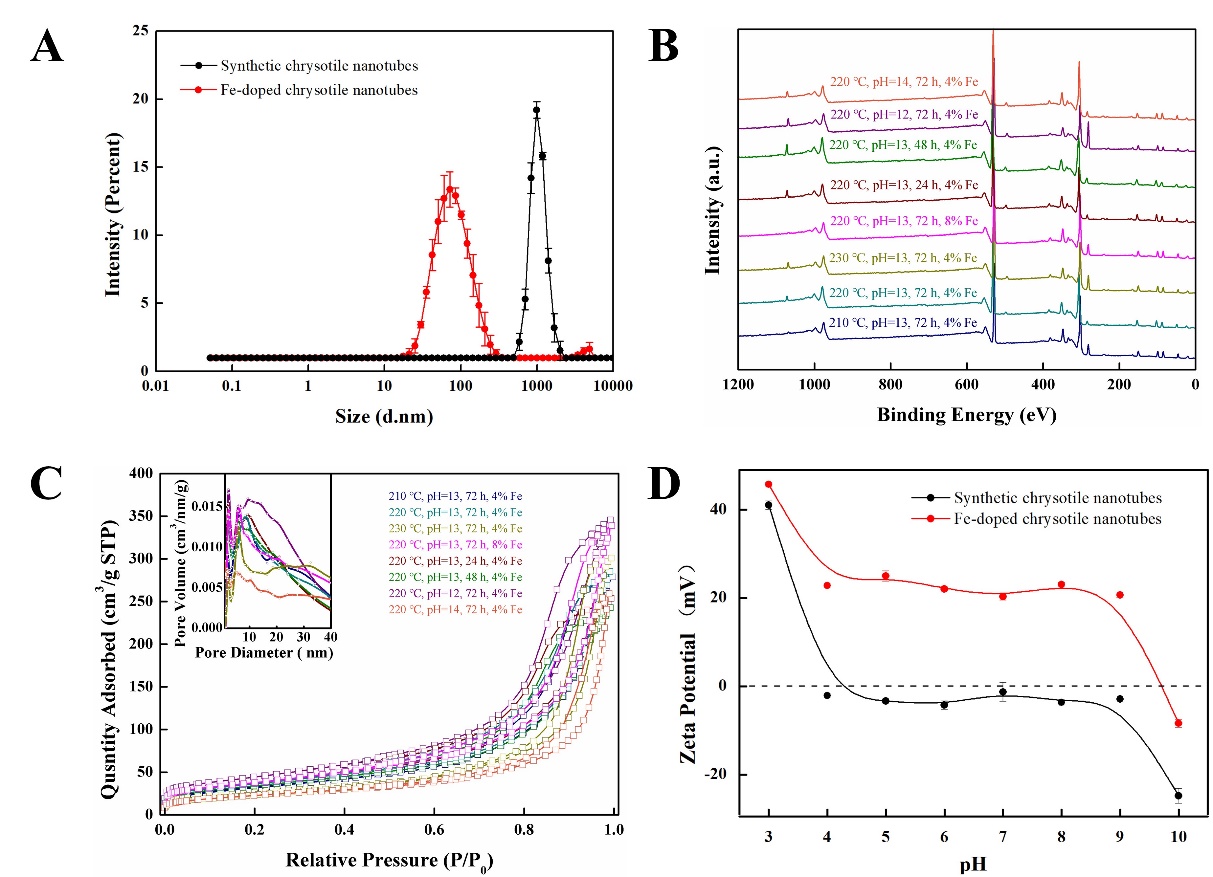
**

**Additional file 3: Figure S3 (A) DSL data, (B) XRD patterns, (C) Nitrogen adsorption-desorption isotherms, and（D）Zeta-potential of FeSiNTs with different hydrothermal environments.**
