# Supplementary material for: Fe-doped chrysotile nanotubes containing siRNAs to silence SPAG5 to treat bladder cancer
Source: J Nanobiotechnology. 2021 Jun 23;19:189. doi: 10.1186/s12951-021-00935-z (PMC8220725; doi:10.1186/s12951-021-00935-z)
Supplement: Supplementary file 4 — Additional file 4: Figure S4. siRNA-binding efficiency (A) and cytotoxicity (B) of FeSiNTs. [file 12951_2021_935_MOESM4_ESM.docx]

**Additional information**


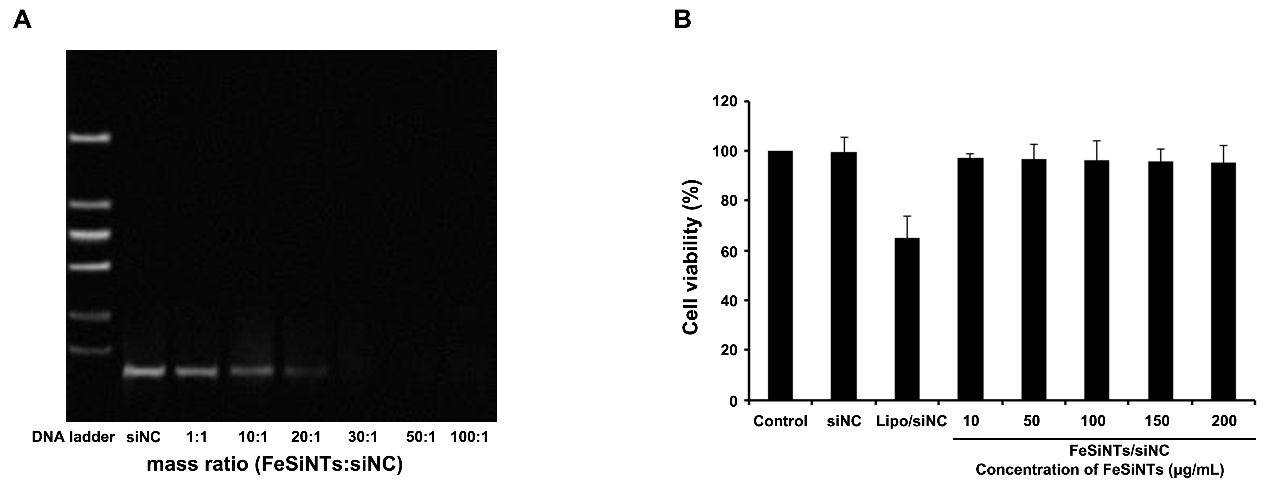


**Additional file 4: Figure S4 siRNA-binding efficiency and cytotoxicity of FeSiNTs.** **(A)** Agarose gel electrophoresis image of different FeSiNTs/siRNA ratios (0, 1, 10, 20, 30, 50, and 100, in mass). **(B)** Cytotoxicity of FeSiNTs detected by CCK-8 assays. No significant change was observed in the growth rate of T24 cells treated with different NPs up to 48 h. Abbreviations: FeSiNTs, Fe doped chrysotile nanotubes; siNC, negative control small interfering RNA; lipo, Lipofectamine 3000.
