# Supplementary material for: Fe-doped chrysotile nanotubes containing siRNAs to silence SPAG5 to treat bladder cancer
Source: J Nanobiotechnology. 2021 Jun 23;19:189. doi: 10.1186/s12951-021-00935-z (PMC8220725; doi:10.1186/s12951-021-00935-z)
Supplement: Supplementary file 5 — Additional file 5: Figure S5. Cytotoxicity of FeSiNTs detected by EdU assays. **P < 0.01. [file 12951_2021_935_MOESM5_ESM.docx]

**Additional information**


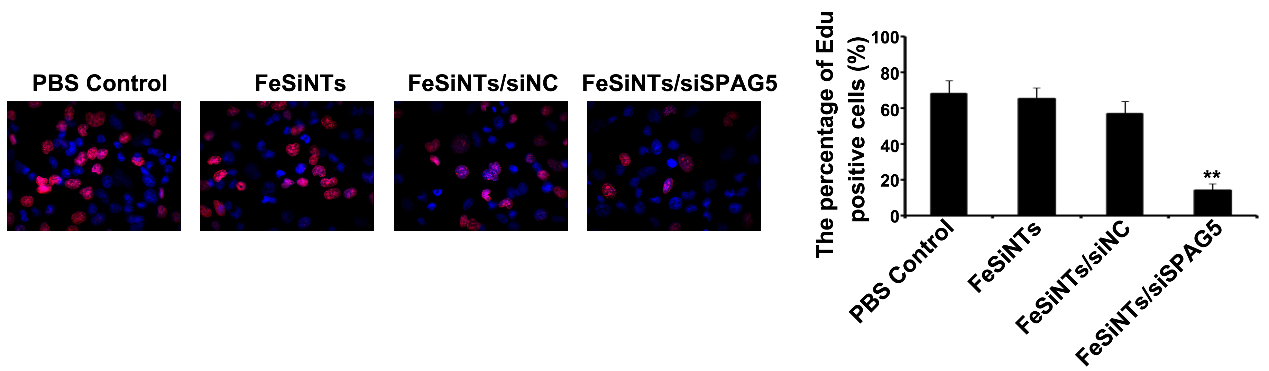


**Additional file 5: Figure S5 Cytotoxicity of FeSiNTs detected by EdU assays.**
