# Supplementary material for: Fe-doped chrysotile nanotubes containing siRNAs to silence SPAG5 to treat bladder cancer
Source: J Nanobiotechnology. 2021 Jun 23;19:189. doi: 10.1186/s12951-021-00935-z (PMC8220725; doi:10.1186/s12951-021-00935-z)
Supplement: Supplementary file 6 — Additional file 6: Figure S6. Relaease analysis of FeSiNTs/siRNA after incubation with medium (A) and serum (B) for different times, as determined by gel electrophoresis. [file 12951_2021_935_MOESM6_ESM.docx]

**Additional information**


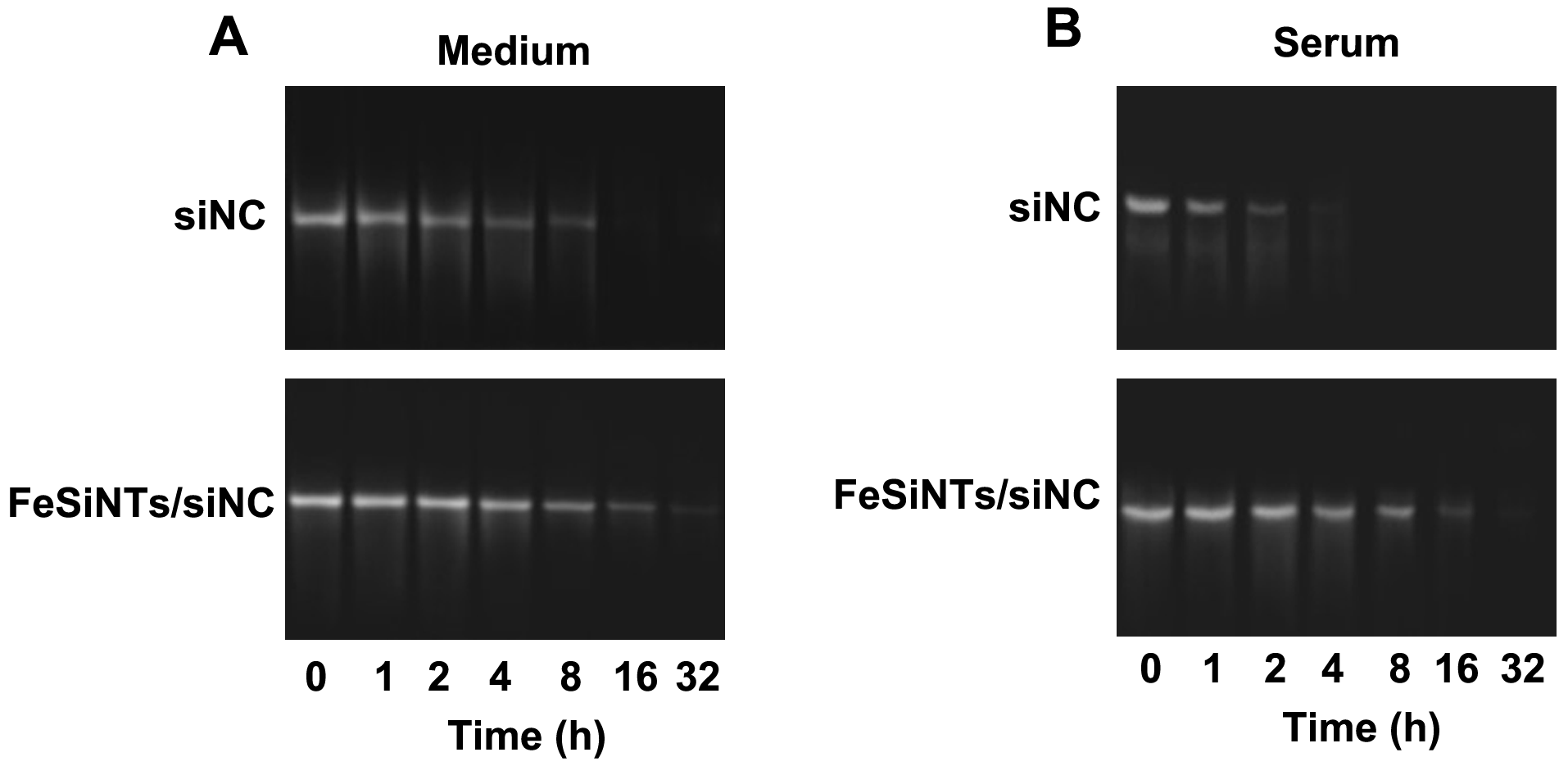


**Additional file 6: Figure S6 Relaease analysis of FeSiNTs/siRNA after incubation with medium (A) and serum (B) for different times, as determined by gel electrophoresis.**
