# Supplementary material for: Fe-doped chrysotile nanotubes containing siRNAs to silence SPAG5 to treat bladder cancer
Source: J Nanobiotechnology. 2021 Jun 23;19:189. doi: 10.1186/s12951-021-00935-z (PMC8220725; doi:10.1186/s12951-021-00935-z)
Supplement: Supplementary file 7 — Additional file 7: Figure S7. Lysosomal escape of FeSiNTs/siSPAG5 in T24 cells. [file 12951_2021_935_MOESM7_ESM.docx]

**Additional information**


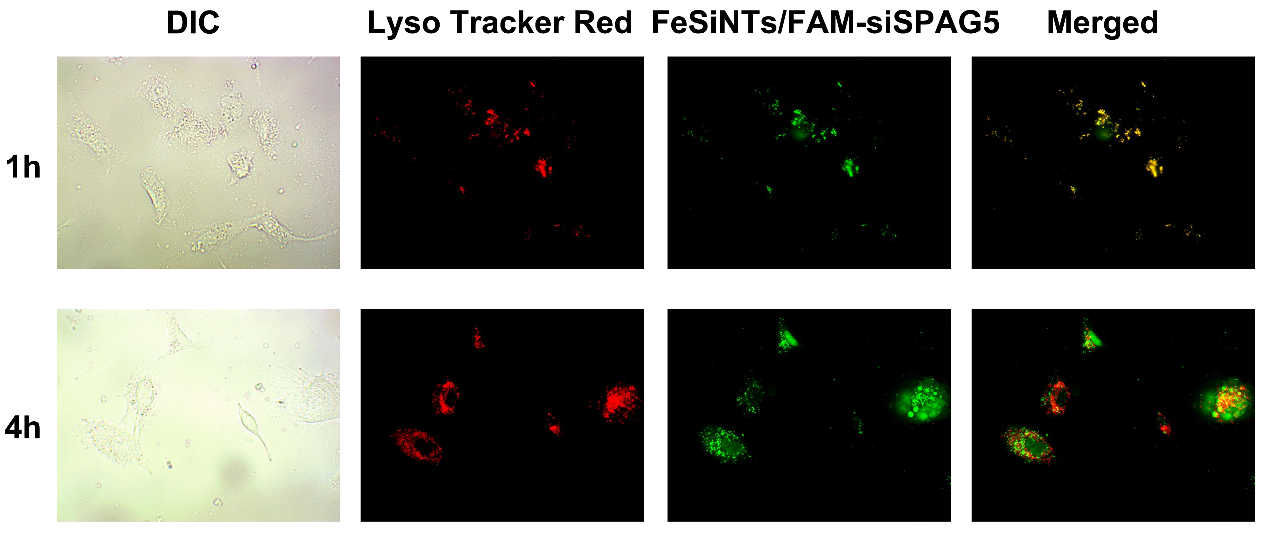


**Additional file 7: Figure S7 Lysosomal escape of FeSiNTs/siSPAG5 in T24 cells.** Confocal images of T24 cells transfected by FeSiNTs/siSPAG5 formulations for 1 h at the final siRNA concentration. siRNA was labeled by FAM (green), endosomes/lysosomes were stained by lysotracker green (red), and merged images are shown. Scale bar is 20 µm.
