# Supplementary material for: Fe-doped chrysotile nanotubes containing siRNAs to silence SPAG5 to treat bladder cancer
Source: J Nanobiotechnology. 2021 Jun 23;19:189. doi: 10.1186/s12951-021-00935-z (PMC8220725; doi:10.1186/s12951-021-00935-z)
Supplement: Supplementary file 8 — Additional file 8: Figure S8. Effects of SPAG5 silencing by FeSiNTs/siSPAG5 on T24 proliferation, detected using a EdU assay. [file 12951_2021_935_MOESM8_ESM.docx]

**Additional information**


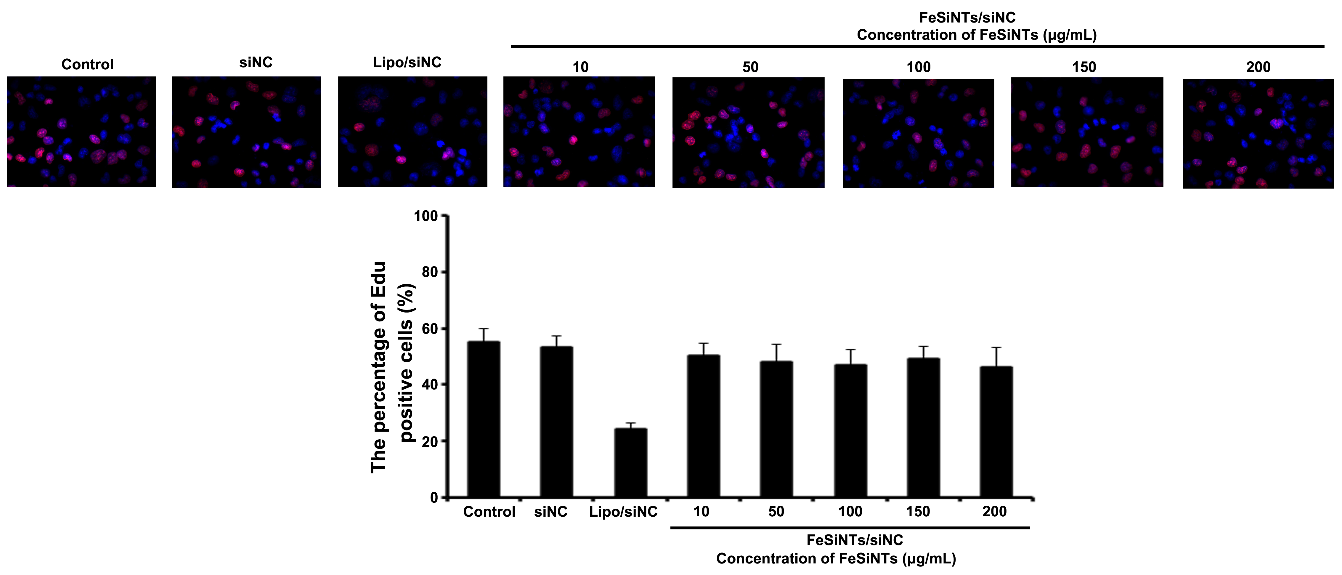


**Additional file 8: Figure S8** Effects of *SPAG5* silencing by FeSiNTs/siSPAG5 on T24 proliferation, detected using a EdU assay.
