# Supplementary material for: Fe-doped chrysotile nanotubes containing siRNAs to silence SPAG5 to treat bladder cancer
Source: J Nanobiotechnology. 2021 Jun 23;19:189. doi: 10.1186/s12951-021-00935-z (PMC8220725; doi:10.1186/s12951-021-00935-z)
Supplement: Supplementary file 9 — Additional file 9: Figure S9. Effects of SPAG5 silencing by FeSiNTs/siSPAG5 on the colony formation ability of T24 cells, tested using a plate colony assay. **P < 0.01. [file 12951_2021_935_MOESM9_ESM.docx]

**Additional information**


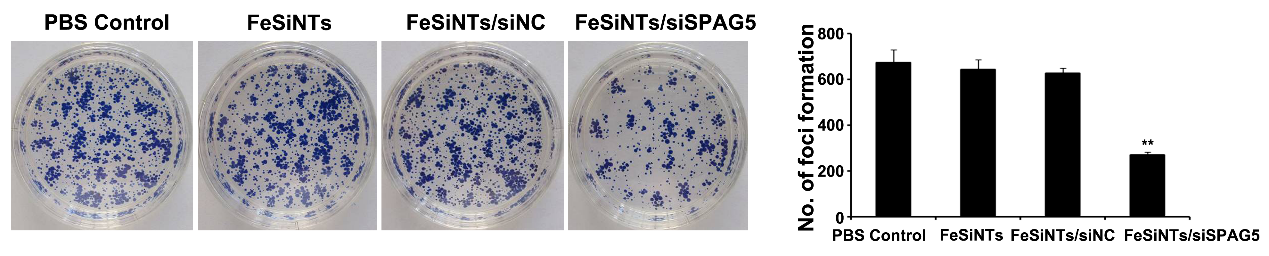


**Additional file 9: Figure S9 Effects of *SPAG5* silencing by FeSiNTs/siSPAG5 on the colony formation ability of T24 cells, tested using a plate colony assay. ***P* < 0.01.**
