# Supplementary material for: Fe-doped chrysotile nanotubes containing siRNAs to silence SPAG5 to treat bladder cancer
Source: J Nanobiotechnology. 2021 Jun 23;19:189. doi: 10.1186/s12951-021-00935-z (PMC8220725; doi:10.1186/s12951-021-00935-z)
Supplement: Supplementary file 10 — Additional file 10: Figure S10. Annexin V and propidium iodide staining of T24 cells treated with FeSiNTs/siSPAG5 complexes and other formulations, and analysis of apoptosis using flow cytometry. **P < 0.01. [file 12951_2021_935_MOESM10_ESM.docx]

**Additional information**


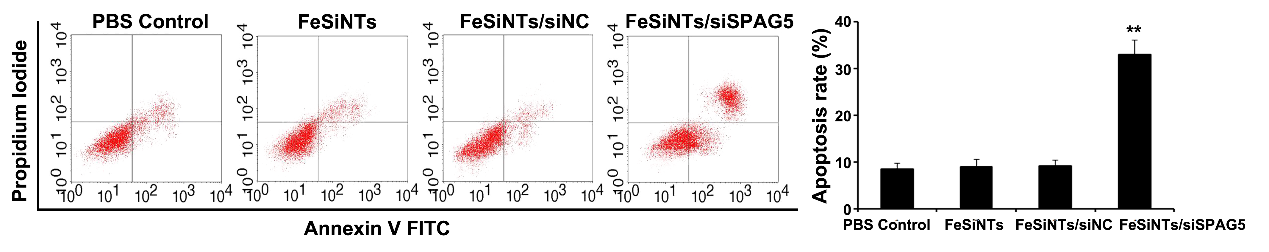


**Additional file 10: Figure S10 Annexin V and propidium iodide staining of T24 cells treated with FeSiNTs/siSPAG5 complexes and other formulations, and analysis of apoptosis using flow cytometry. ***P* < 0.01.**
