# Supplementary material for: Fe-doped chrysotile nanotubes containing siRNAs to silence SPAG5 to treat bladder cancer
Source: J Nanobiotechnology. 2021 Jun 23;19:189. doi: 10.1186/s12951-021-00935-z (PMC8220725; doi:10.1186/s12951-021-00935-z)
Supplement: Supplementary file 11 — Additional file 11: Figure S11. After FeSiNTs-mediated SPAG5 knockdown in T24 cells, FACS was used to analyze the cell cycle. **P < 0.01. [file 12951_2021_935_MOESM11_ESM.docx]

**Additional information**


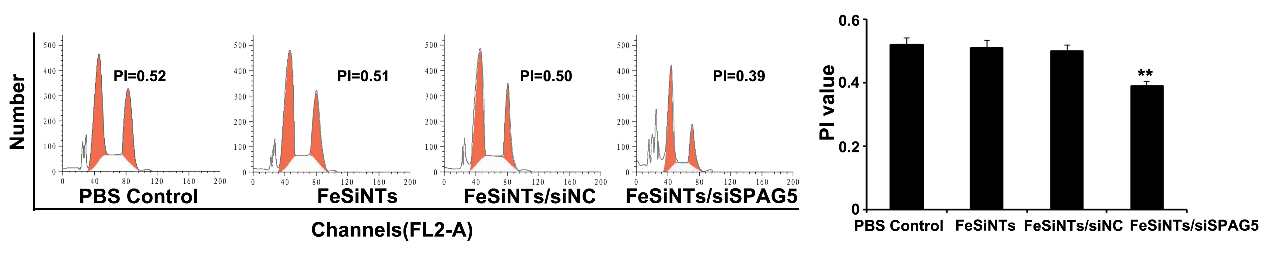


**Additional file 11: Figure S11 After FeSiNTs-mediated *SPAG5* knockdown in T24 cells, FACS was used to analyze the cell cycle. ***P* < 0.01.**
