# Supplementary material for: Fe-doped chrysotile nanotubes containing siRNAs to silence SPAG5 to treat bladder cancer
Source: J Nanobiotechnology. 2021 Jun 23;19:189. doi: 10.1186/s12951-021-00935-z (PMC8220725; doi:10.1186/s12951-021-00935-z)
Supplement: Supplementary file 12 — Additional file 12: Figure S12. Tumour tissues analyzed using western blotting from the subcutaneous xenograft model. [file 12951_2021_935_MOESM12_ESM.docx]

**Additional information**


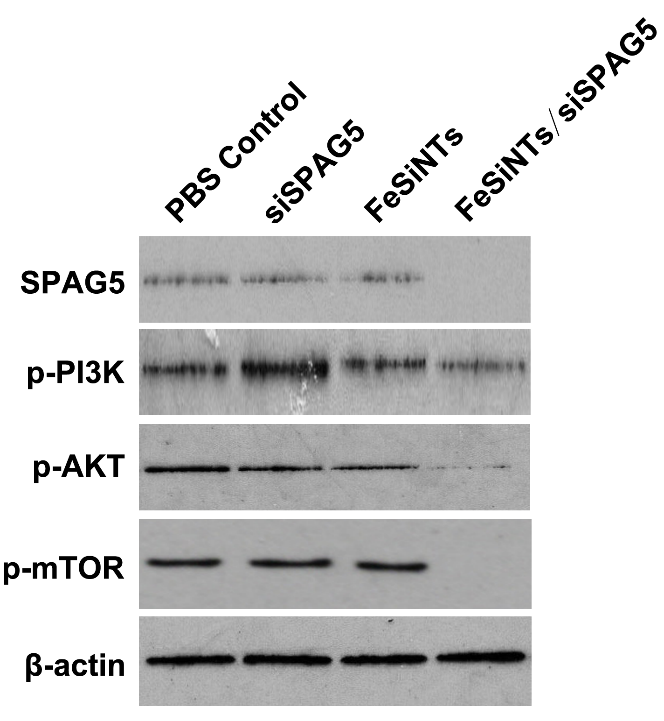


**Additional file 12: Figure S12 Tumour tissues analyzed using western blotting from the subcutaneous xenograft model.** Tissue lysates analyzed using western blot with anti-SPAG5, anti‑PI3K, anti-p-AKT, anti-p-mTOR, and anti-β-actin antibodies.
