# Supplementary material for: Fe-doped chrysotile nanotubes containing siRNAs to silence SPAG5 to treat bladder cancer
Source: J Nanobiotechnology. 2021 Jun 23;19:189. doi: 10.1186/s12951-021-00935-z (PMC8220725; doi:10.1186/s12951-021-00935-z)
Supplement: Supplementary file 13 — Additional file 13: Figure S13. SPAG5 and Ki67 immunohistochemistry analysis, and TUNEL staining analysis of the tumors treated with PBS, siSPAG5, FeSiNTs, and FeSiNTs/siSPAG5. **P < 0.01. [file 12951_2021_935_MOESM13_ESM.docx]

**Additional information**


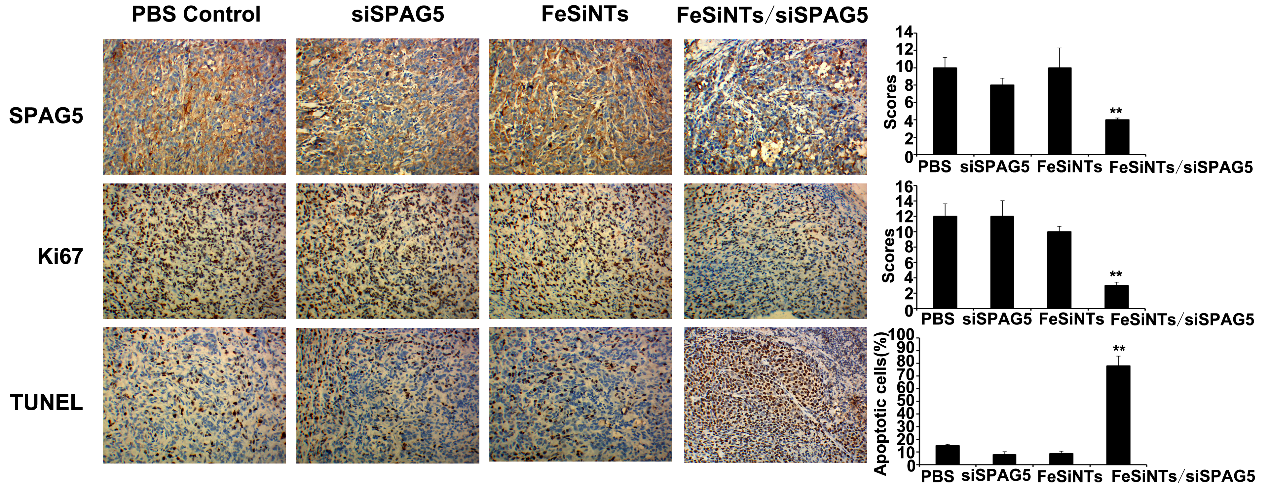


**Additional file 13: Figure S13 SPAG5 and Ki67 immunohistochemistry analysis, and TUNEL staining analysis of the tumors treated with PBS, siSPAG5, FeSiNTs, and FeSiNTs/siSPAG5. ***P* < 0.01.**
