# Supplementary material for: Fe-doped chrysotile nanotubes containing siRNAs to silence SPAG5 to treat bladder cancer
Source: J Nanobiotechnology. 2021 Jun 23;19:189. doi: 10.1186/s12951-021-00935-z (PMC8220725; doi:10.1186/s12951-021-00935-z)
Supplement: Supplementary file 14 — Additional file 14: Table S1. Tumor suppression effect of different treatment on bladder histopathologic changes in SD rat bladders of different groups. [file 12951_2021_935_MOESM14_ESM.docx]

**Additional information**

| **Additional file 14: Table S1** Tumor suppression effect of different treatment on bladder histopathologic changes in SD rat bladders of different groups. | | | | | |
| --- | --- | --- | --- | --- | --- |
| Group | No.of rats sacrificed | Normal (T0) | Cancer in situ or Noninvasive papillary carcinoma (Tis/Ta) | Subepithelial connective tissue invasive bladder cancer (T1) | Muscle invasive bladder cancer  (≥T2) |
| PBS Control | 15 | 2 | 2 | 1 | 10 |
| siSPAG5 | 15 | 1 | 2 | 1 | 11 |
| FeSiNTs | 15 | 1 | 3 | 2 | 9 |
| FeSiNTs/siSPAG5 | 15 | 4 | 7 | 3 | 1 |
|  |  |  |  |  |  |
