# Supplementary material for: Fe-doped chrysotile nanotubes containing siRNAs to silence SPAG5 to treat bladder cancer
Source: J Nanobiotechnology. 2021 Jun 23;19:189. doi: 10.1186/s12951-021-00935-z (PMC8220725; doi:10.1186/s12951-021-00935-z)
Supplement: Supplementary file 15 — Additional file 15: Figure S14. Biosafety evaluation of FeSiNTs/siSPAG5 in vivo. [file 12951_2021_935_MOESM15_ESM.docx]

**Additional information**


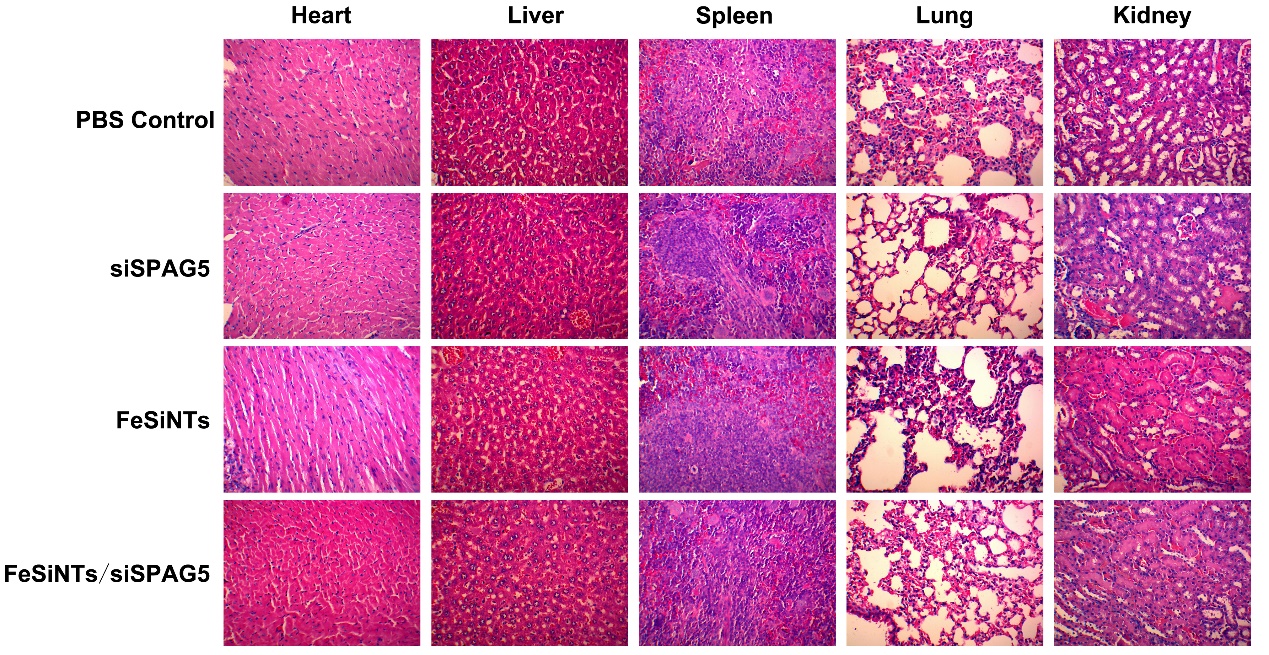


**Additional file 15: Figure S14 Biosafety evaluation of FeSiNTs/siSPAG5 *in vivo*.** Histopathological analyses of H&E-stained tissue sections from the heart, liver, spleen, lung, and kidney of tumor-bearing mice after the indicated treatments, scale bar: 20 μm.
