# Supplementary material for: Fe-doped chrysotile nanotubes containing siRNAs to silence SPAG5 to treat bladder cancer
Source: J Nanobiotechnology. 2021 Jun 23;19:189. doi: 10.1186/s12951-021-00935-z (PMC8220725; doi:10.1186/s12951-021-00935-z)
Supplement: Supplementary file 16 — Additional file 16: Table S2. Biochemical parameters of blood from the mice at 24 h after last injection of various drugs [file 12951_2021_935_MOESM16_ESM.docx]

**Additional information**

| **Additional file 16: Table S2** Biochemical parameters of blood from the mice at 24 h after last injection of various drugs. | | | | |
| --- | --- | --- | --- | --- |
| Group | ALT (U/L) | AST (U/L) | BUN (nM) | CREA (μM) |
| PBS Control | 65±13 | 187±16 | 5.2±1.1 | 36±4 |
| siSPAG5 | 97±16 | 198±20 | 6.4±0.8 | 45±3 |
| FeSiNTs | 68±15 | 106±12 | 7.5±1.5 | 30±2 |
| FeSiNTs/siSPAG5 | 75±21 | 143±17 | 6.7±1.4 | 32±3 |
| Reference | 17-132 | 54-298 | 2.8-11.7 | 18-80 |
