# Supplementary material for: Fe-doped chrysotile nanotubes containing siRNAs to silence SPAG5 to treat bladder cancer
Source: J Nanobiotechnology. 2021 Jun 23;19:189. doi: 10.1186/s12951-021-00935-z (PMC8220725; doi:10.1186/s12951-021-00935-z)
Supplement: Supplementary file 17 — Additional file 17: Figure S15. IFN-α (A), IL-1β (B), IL-6 (C), and TNF-α (D) analysis of mouse blood at 24 h after PBS, siSPAG5, FeSiNTs, and FeSiNTs/siSPAG5 injection. [file 12951_2021_935_MOESM17_ESM.docx]

**Additional information**


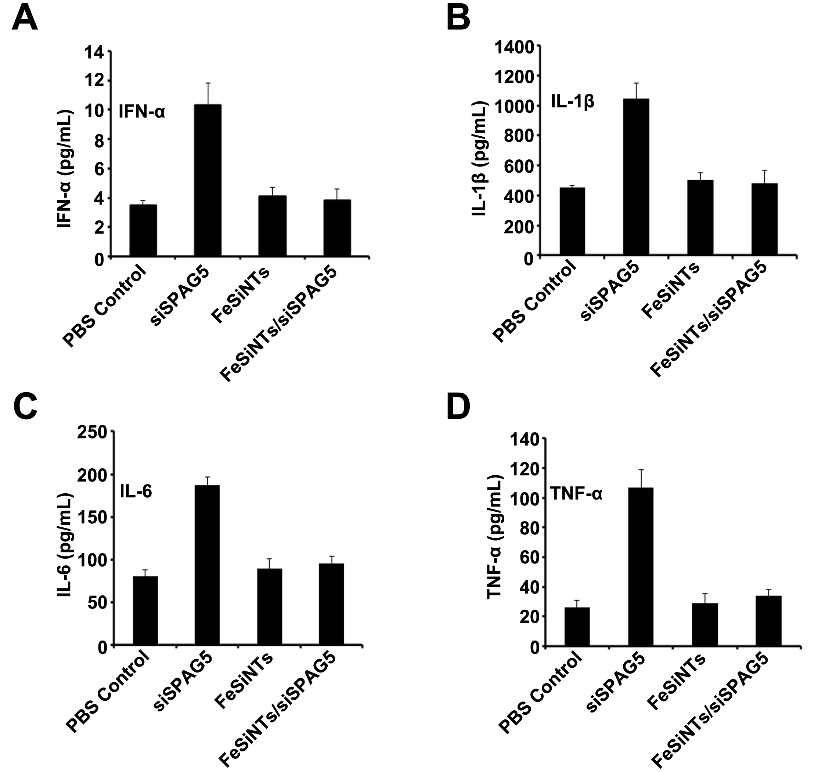


**Additional file 17: Figure S15 Immunotoxicity analysis of mouse blood at 24 h after PBS, siSPAG5, FeSiNTs, and FeSiNTs/siSPAG5 injection.** IFN-α **(A)**, IL-1β **(B)**, interleukin-6 (IL-6) **(C)**, and tumor necrosis factor-a (TNF-a) **(D)** levels were determined using ELISA.
