# Supplementary material for: Fe-doped chrysotile nanotubes containing siRNAs to silence SPAG5 to treat bladder cancer
Source: J Nanobiotechnology. 2021 Jun 23;19:189. doi: 10.1186/s12951-021-00935-z (PMC8220725; doi:10.1186/s12951-021-00935-z)
Supplement: Supplementary file 18 — Additional file 18: Figure S16. Immunohistochemistry analysis of members of the PI3K/AKT/mTOR signaling pathway in tumor tissues treated with PBS, siSPAG5, FeSiNTs, and FeSiNTs/siSPAG5. **P < 0.01. [file 12951_2021_935_MOESM18_ESM.docx]

**Additional information**


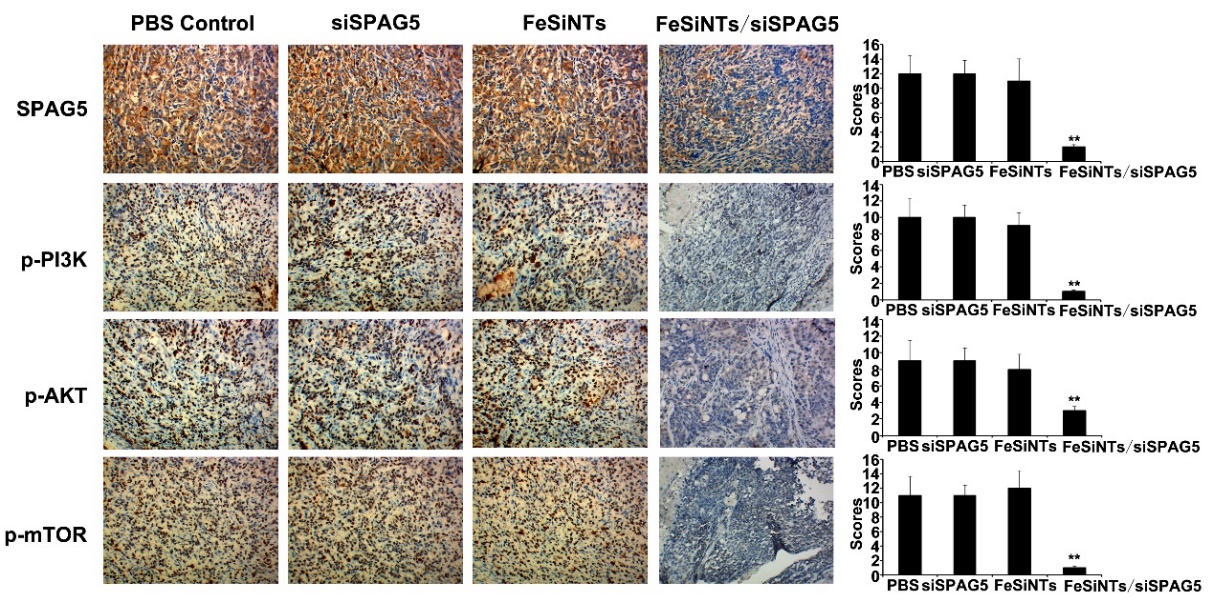


**Additional file 18: Figure S16 Immunohistochemistry analysis of members of the PI3K/AKT/mTOR signaling pathway in tumor tissues treated with PBS, siSPAG5, FeSiNTs, and FeSiNTs/siSPAG5. ***P* < 0.01.**
